# Longitudinal changes in the cystic fibrosis airway microbiota with time and treatment

**DOI:** 10.1016/j.jcf.2023.11.010

**Published:** 2023-12-28

**Authors:** Gisli G. Einarsson, Laura J. Sherrard, Joseph E. Hatch, Bryan Zorn, Elinor Johnston, Clodagh McGettigan, Katherine O’Neill, Deirdre F. Gilpin, Damian G. Downey, Michelle Murray, Gillian Lavelle, Gerry McElvaney, Matthew C. Wolfgang, Richard Boucher, Marianne S. Muhlebach, Ian Bradbury, J. Stuart Elborn, Michael M. Tunney

**Affiliations:** a Queen’s University Belfast, Belfast, United Kingdom; b Royal College of Surgeons in Ireland, Beaumont Hospital, Dublin, Ireland; c University of North Carolina, Chapel Hill, NC, United States; d Frontier Science (Scotland) Ltd., Kincraig, United Kingdom

**Keywords:** Microbiota, Respiratory infections, Exacerbations, Pseudomonas aeruginosa

## Abstract

**Background:**

Whether there is any benefit in integrating culture-independent molecular analysis of the lower airway microbiota of people with cystic fibrosis into clinical care is unclear. This study determined the longitudinal trajectory of the microbiota and if there were microbiota characteristics that corresponded with response to treatment or predicted a future pulmonary exacerbation.

**Methods:**

At least one sputum sample was collected from 149 participants enrolled in this prospective longitudinal multi-centre study and total bacterial density and microbiota community measurements were determined and compared with clinical parameters.

**Results:**

In 114 participants with paired samples when clinically stable, ~8 months apart, the microbiota remained conserved between timepoints, regardless of whether participants received acute intravenous antibiotic treatment or not. In 62 participants, who presented with an acute exacerbation, a decrease in community richness correlated best with patient response to antibiotic treatment. Analysis of baseline samples from 30 participants who exacerbated within 4 months of their stable sample being collected and 72 participants who remained stable throughout the study showed that community characteristics such as lower richness at baseline may be predictive of an exacerbation in addition to several clinical parameters. However, lasso regression analysis indicated that only lung function (*p* = 0.014) was associated with a future exacerbation.

**Conclusions:**

The airway microbiota remains stable over periods <1 year with modest shifts related to treatment apparent which might provide some additional insights to patient-level measurements.

## 1. Introduction

Chronic respiratory infection is the primary cause of morbidity and mortality in people with cystic fibrosis (PWCF) with repeated acute pulmonary exacerbations associated with a decline in lung function and an increased requirement for antimicrobial therapy [1]. Routine aerobic culture-based approaches detect a small number of organisms such as *Staphylococcus aureus, Pseudomonas aeruginosa, Burkholderia cepacian* complex and *Stenotrophomonas maltophilia* as the principal pathogenic drivers of infection and inflammation in the CF airways [1]. However, many culture-independent studies have revealed the presence of a much more complex polymicrobial airway microbiota with multiple obligate anaerobic genera frequently detected [2-4]. Ecological analysis of such studies has generally reported that, as the airway microbiota evolves over time, there is reduced bacterial diversity and increased dominance of recognised CF pathogens, which is associated with decreased lung function, increased antibiotic use and age [2-7].

Conventionally, pulmonary exacerbations were thought to result from an increase in bacterial burden or acquisition of a new strain of a recognised CF pathogen followed by an elevated host-inflammatory response [8,9]. However, a number of studies have demonstrated that the onset of a pulmonary exacerbation is not solely due to these factors and the underlying causes are often unknown [10-12]. For example, some studies investigating the role of the airway microbiota in pulmonary exacerbations have reported that variation in community contribution, by members other than recognised CF pathogens, might be important [13-16].

A key contemporary consideration for CF is how complex airway microbiota data could augment current patient care including use as a surrogate biomarker to indicate disease progression, monitor response to treatment and/or predict the onset of a pulmonary exacerbation [7,14,17].

In this prospective study carried out in three CF centres, we used quantitative polymerase chain reaction (qPCR) and high-throughput targeted amplicon sequencing to characterise the total bacterial density and microbiota composition and structure in sputum samples collected longitudinally from PWCF. While total bacterial density can be determined by quantitative culture methods, it is not standard practice but could be ‘easily’ integrated using qPCR. Assessments of the microbiota composition and structure are more complex and currently require bioinformatic expertise to implement. Here we, firstly, determined the trajectory of the lower airway microbiota communities in PWCF over an 8 month period. Then, we assessed whether the sputum microbial density and/or diversity metrics provide additional valuable information to clinical investigations in two situations: response to treatment of and/or to predict future episodes of a pulmonary exacerbation.

## 2. Methods

### 2.1. Participants and sample collection

PWCF [18] attending CF centres in Belfast in the UK (Adult and Paediatric CF Centres, Belfast Health and Social Care Trust), Dublin in Ireland (Beaumont Hospital and Our Lady’s Children’s Hospital, Crumlin) and University of North Carolina (UNC) at Chapel Hill, NC in the USA (UNC Hospital Pediatric and Adult Clinics) were enrolled into the study at routine outpatient appointments between July 2010 and November 2013. Ethical approval for the study was obtained at each institution, and informed consent/assent was obtained from all adults, parents and paediatric subjects. Studies involving the participants recruited have been previously published [6,10,19-21].

Participants were included in this study if they spontaneously expectorated sputum. The collection of sputum samples has been described previously [6,10]. Briefly, sputum was collected from participants at study enrolment during clinical stability, which was defined as no requirement for intravenous (IV) and/or inhaled or oral antibiotics for respiratory associated symptoms in the prior four weeks. Up to two additional stable samples were collected at outpatient appointments, usually 4 months apart, from participants who remained clinically stable (no requirement for IV antibiotics) during the study. For participants who presented with a pulmonary exacerbation as defined by Fuchs et al., [22] and after collection of at >1 stable sample, sputa were collected at the onset of and completion of IV antibiotic treatment [23]. A further stable sample was collected 4–8 weeks post-exacerbation at outpatient review.

### 2.2. Clinical data

Demographics and medical information were collected for each participant, including age, gender, cystic fibrosis transmembrane conductance regulator (CFTR) function, [24] body mass index (BMI), spirometry (forced expiratory volume in one-second [FEV_1_] % predicted), co-morbidities, long-term medical treatments prescribed, and IV antibiotics used to treat pulmonary exacerbations.

### 2.3. Sample preparation for molecular analysis

Total genomic DNA (gDNA) was extracted from ~200 mg sputum aliquots, along with technical controls, by treatment with Sputolysin^®^, mechanical disruption and processing on an automated MagNA Pure nucleic acid purification platform (Roche Diagnostics, Indianapolis, USA). Following gDNA extraction, template clean-up and assessment of template concentration using the LVis Microplate on the FLUOstar Omega Microplate Reader (BMG LABTECH, Aylesbury, UK) was completed. Samples were stored at −20 °C until further use.

### 2.4. Quantification of total bacterial (16S rRNA) density

Total bacterial density (copy number per mL; copies/mL) was determined using a primer/probe targeting the bacterial 16S rRNA marker-gene as previously described, [25] and performed by qPCR on the LightCycler^®^480 instrument (Roche Diagnostics, Mannheim, DE) using the Probes Master kit.

### 2.5. Illumina MiSeq sequencing and data pre-processing

The Illumina MiSeq next-generation sequencing platform (Illumina, USA) targeting the 16S rRNA marker gene was used as previously described to determine microbiota composition and structure [26]. In brief, we targeted the V4-region in a two-step library preparation, applying modified universal primers as previously described by Lundberg et al [27]. Bead-cleaned nucleotide libraries in equimolar concentrations of amplicons (~452 base pairs in size) were sequenced on the platform using the version-3 (V3) paired-end 600 cycle kit. Raw sequence data was deposited to the NCBI-SRA under Bioproject Accession Number PRJNA865935.

Pre-processing of sequence data for downstream analysis of raw sequence read, operational taxonomic units (OTUs) calling and data analysis was performed in Quantitative Insights Into Microbial Ecology (QIIME) version 1.9.1 [28]. Additional details on sample handling, MiSeq library preparation, quantification, 16S rRNA marker-gene sequencing, handling and removal of potential background contamination observed in technical sequencing controls and analysis are provided in the online supplement (including Table S1). The metadata mapping file for MiSeq processing and analysis is provided in the online supplement (file S1).

### 2.6. Statistical analysis and microbiota community measurements

Demographics and clinical information of participants recruited at the three CF centres were compared using a Kruskal-Wallis test or Pearson’s chi-squared test, as appropriate. A Wilcoxon-signed rank test was used to compare related patient data between time-points.

A detailed description of the assessment of microbiota community data is provided in the online supplement. Briefly, taxa abundances are shown as relative abundance. The alpha-diversity (within group) indices, taxonomic richness [S], diversity (Shannon-Wiener Index [H], evenness [e^H/S^] and dominance [D], were calculated. Beta-diversity (between analytical groups) was assessed and presented as a principle coordinates plot (PCoA) showing variance explained for the first two components. Differences between groups were evaluated by multivariate-permutational analysis (PERMANOVA) as implemented within the adonis test with 9999 permutations.

To determine factors which could potentially be used to predict a future pulmonary exacerbation, participants were categorised as either cases (exacerbated within 4 months of a previous stable sample; *n* = 30) or controls (no evidence of an exacerbation during the study and time between study visits was >4 months; *n* = 72) and baseline co-variates were compared (adjusted odds ratios and 95% confidence intervials are reported). In our cohort, 4 months was chosen as the cut-off to reflect the median duration between outpatient appointments, when patients are clinically stable [10]. A lasso (least absolute shrinkage and selection operator) regression was then used to assess the impact of the covariates on the outcome of a future pulmonary exacerbation. A sensitivity analysis was also performed with alternative cut-off periods (3 and 6 months) in the lasso regression.

Data analyses were performed in R version 4.1.2 and IBM SPSS Statistics version 26. *p*<0.05 was accepted as statistically significant.

## 3. Results

### 3.1. Cohort characteristics

At least one sputum sample was collected from 149 study participants. The overall demographics and clinical characteristics of the cohort at study enrolment are shown in Table 1. CF centre-specific differences in participants were observed (Table 1) as described previously [29]. For example, the Belfast cohort was older but had features of a milder disease phenotype. No patients took CFTR modulators during the study period.

**Fig. 1.**
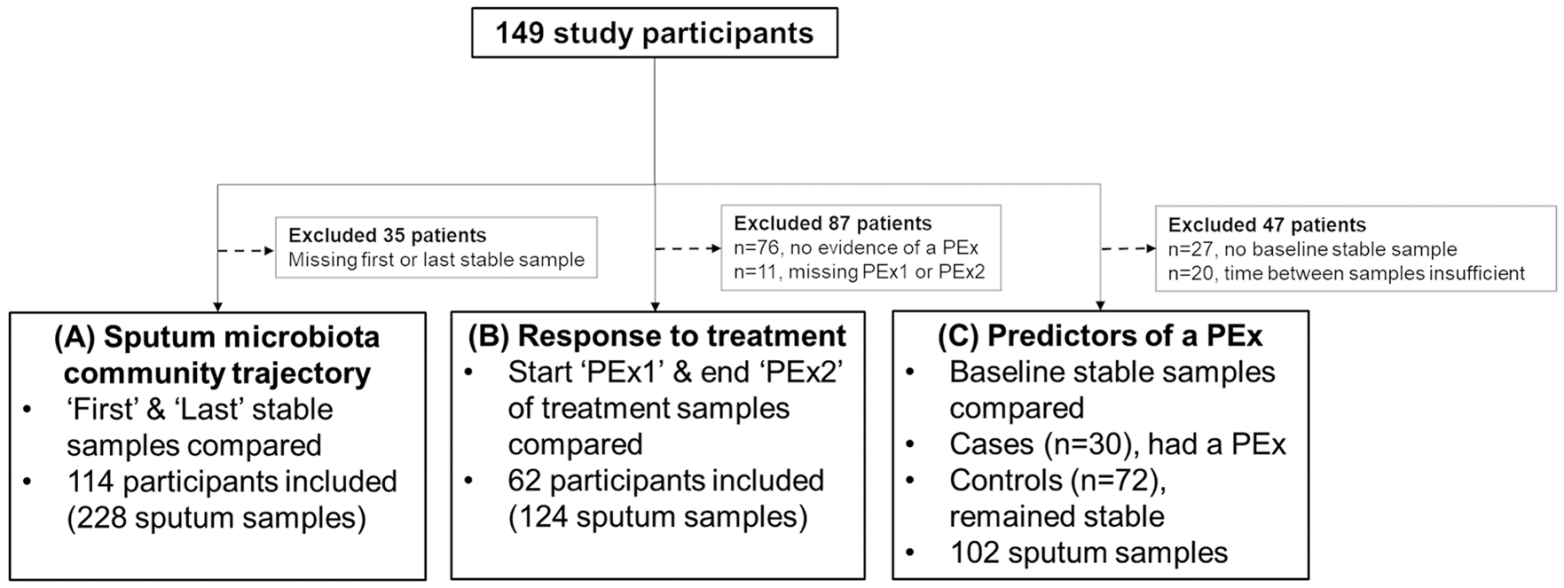
Patient stratification to address the study objectives. **(A)** Determine the trajectory of the lower airway microbiota communities in PWCF over an 8 month period. Paired stable sputa samples (‘First’ and ‘Last’) collected at outpatient appointments were compared. **(B)** Assess if there are microbiota community characteristics that correspond with response to treatment of a pulmonary exacerbation (PEx). Paired samples collected at the onset (‘PEx1’) and completion (‘PEx2’) of treatment, when the patient had recovered, were compared. **(C)** Assess if there are microbiota community characteristics that are able to predict future PEx episodes. Participants were categorised as either cases (exacerbated within 4 months of previous stable sample) or controls (no evidence of an exacerbation during the study and the time between study visits was >4 months) and baseline stable sputum community characteristics were compared.

Fig. 1 shows the stratification of participants to address the study objectives.

### 3.2. Microbiota community trajectory with time

To determine if community composition characteristics changed with time between periods of clinical stability, paired stable sputa samples (‘First’ and ‘Last’) collected at outpatient appointments were compared.

In total, 114 paired samples (study site: Belfast, *n* = 73; Dublin, *n* = 8; Chapel Hill, *n* = 33) were available, with a mean of 35 weeks (~8 months) (standard deviation [SD], 18 weeks) between outpatient appointments. This cohort had a mean age of 27.6 years and 50 (43.9%) participants were female. Overall, 24.5% of participants had mild lung disease (FEV_1_: >80 %predicted), 63.2% had moderate lung disease (FEV_1_: 40–80 %predicted) and 12.3% had advanced disease (FEV_1_: <40 %predicted). There was no significant difference in lung function (mean FEV_1_; 66.1 %predicted [First] vs. 65.3%predicted [Last]; *p* = 0.791) or BMI (22.2 kg/m^2^ [First] vs. 22.4 kg/m^2^ [Last]; *p* = 0.807) between timepoints.

**Fig. 2.**
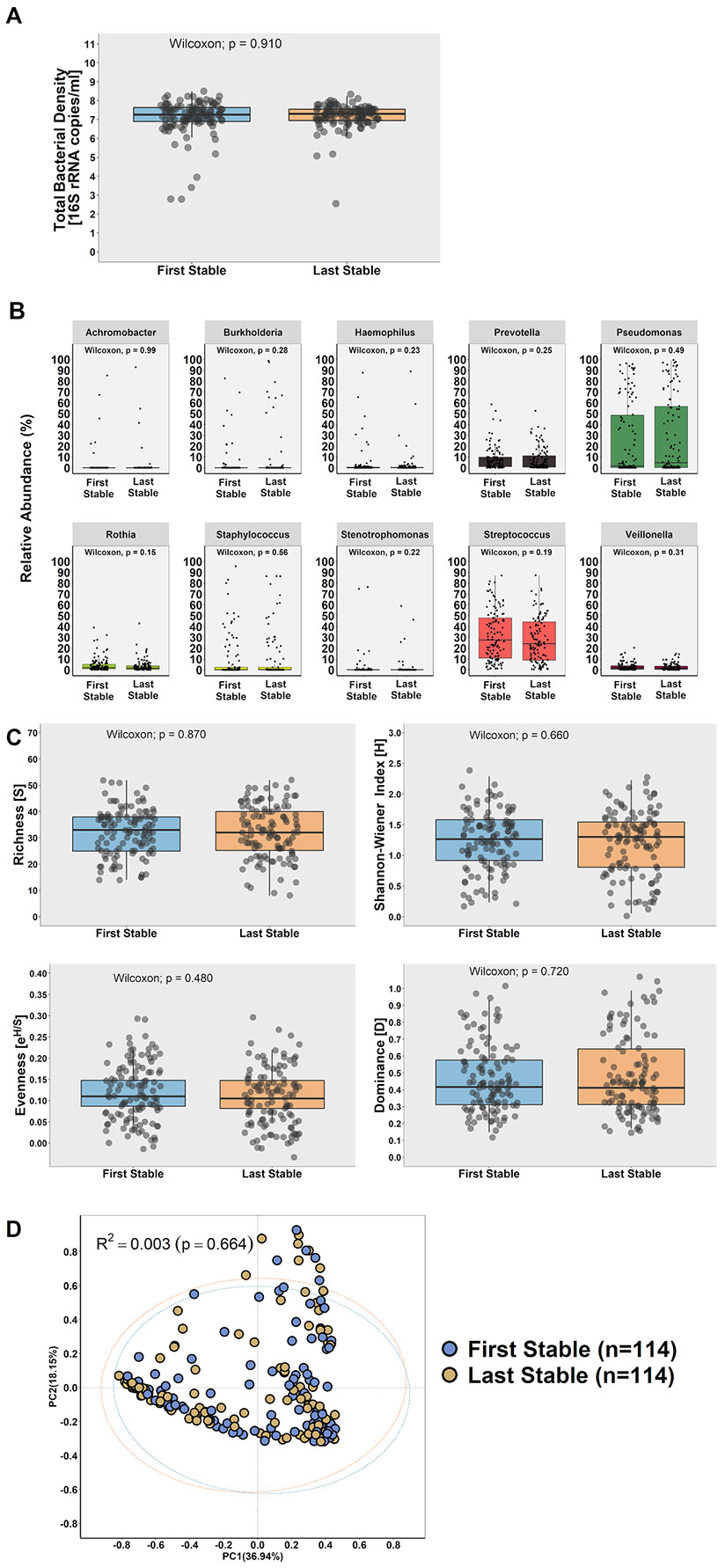
Comparison of microbial community characteristics between the ‘First’ and ‘Last’ sputum samples collected at outpatient appointments when participants (*n* = 114) were clinically stable. (A) Total bacterial density quantification by qPCR corresponding to the total number of 16S rRNA encoding gene copies per mL of sputum. (B) The top 10 genera detected. (C) Ecological parameters, taxonomic richness [S], community diversity (Shannon Wiener Index [H]), evenness [e^H/S^] and dominance [D]. In the box and whisker plots in figures A-C, the line inside the box indicates the median and the top and bottom of the box indicate the 25th and 75th percentile, respectively. The whiskers indicate the 90% confidence interval (CI). Samples were compared using the Wilcoxon-signed rank test. (D) Principal coordinate analysis (PCoA) plot comparing microbial communities based on the adonis analysis with Hellinger transformed data and Bray-Curtis distance (R^2^=0.003, *p* = 0.664, 9999 permutations; confidence based on 90% CI).

Although some intra-participant variation in microbiota community composition was detected in paired samples (Fig. S1), overall, no difference was observed in the total bacterial density (7.26 [6.90–7.64] copies/mL [First: median; 25th and 75th percentiles; Log10] vs. 7.30 [6.95–7.55] copies/mL [Last: median; 25th and 75th percentiles; Log10]; *p* = 0.910; Fig. 2A) nor in the relative abundance of detected taxa, including the 10 most prevalent genera (*p*>0.05) (Fig. 2B). In addition, no significant differences (*p*>0.05) were observed for the main alpha-diversity indices between the sampling time-points (Fig. 2C). Likewise, no significant difference in beta-diversity (community dispersal) was observed between the time-points, with the microbiota accounting for ~0.3% of the observed community variance (adonis analysis with Hellinger transformed data and Bray-Curtis distance, R^2^=0.003, *p* = 0.664, 9999 permutations). This is depicted visually in the PCoA plot which indicated near complete overlap of the communities (Fig. 2D).

Similarly, no significant differences were found for total bacterial density (*p*>0.05) (Figs. S2A and S3A) and alpha-diversity indices (*p*>0.05) (Figs. S2B and S3B) when participants were stratified based on their pulmonary exacerbation status (exacerbated, *n* = 40; remained stable, *n* = 74) or by length of time (<25 weeks, *n* = 35; 25–38 weeks, *n* = 40; >39 weeks, *n* = 39) between collection of the First and Last stable samples. Both pulmonary exacerbation status and time had a significant effect on beta-diversity; however, this only accounted for ~1% of the explained variance in either case (adonis analysis with Hellinger transformed data and Bray-Curtis distance, R^2^=0.010, *p* = 0.034, 9999 permutations) (Figs. S2C and S3C).

Other variables that may contribute to microbial community structure were also considered. Study site but not stable sample visit (First or Last) had a modest statistically significant effect on community structure which accounted for 2.7% of the explained variance (Fig. S4; R^2^=0.027, *p* = 0.002). Higher community richness [S] and diversity (Shannon-Wiener Index [H]) were found when taxa such as *Streptococcus* spp. and strict anaerobes (e.g., *Prevotella* spp. and *Veillonella* spp.) were present and *Pseudomonas* spp. or *Staphylococcus* spp. were present in low abundance (Fig. S4). Conversely, community dominance [D] and use of inhaled antibiotics and chronic oral azithromycin were associated with communities that had a higher relative abundance of *Pseudomonas* spp. (Fig. S4).

Participants were then segregated into two groups depending on the relative abundance of *Pseudomonas* spp. in the First stable sample (relative abundance: ≥75%, *n* = 15; <75%, *n* = 99), given that *P. aeruginosa* is the most common pathogen in PWCF, and the microbiota characteristics were compared between the First and Last stable visits. Differences were observed for all alpha-diversity indices between the paired samples in those with ≥75% relative abundance *Pseudomonas* spp. while no differences were observed in the microbiota of those with <75% relative abundance (Figs. S5A and S5B). A significant difference was also observed in beta-diversity with *Pseudomonas* spp. relative abundance groups accounting for ~1.1% of the variance explained (adonis analysis with Hellinger transformed data and Bray-Curtis distance, R^2^=0.011; *p* = 0.044, 9999 permutations) (Fig. S5C).

### 3.3. Microbiota community characteristics and their association with response to treatment of a pulmonary exacerbation

To determine microbiota community characteristics that correlated with response to acute antibiotic treatment of a pulmonary exacerbation, we compared paired samples collected at the onset (‘PEx1’) and completion (‘PEx2’) of treatment when the patient had recovered. A total of 62 participants were eligible for inclusion in this analysis (study site: Belfast, *n* = 35; Dublin, *n* = 15; Chapel Hill, *n* = 12) and the mean (SD) time between the study timepoints was 17.3 (9.74) days. Diverse regimens were used for acute treatment but the majority of regimens contained at least one antipseudomonal antibiotic with IV tobramycin in combination with either piperacillin/tazobactam, meropenem or ceftazidime most common. This cohort had a mean age of 25.9 years and 35 (56.5%) participants were female. Lung function significantly improved (mean FEV_1_: 47.2 %predicted [PEx1] vs. 55.0%predicted [PEx2]; *p* = 0.046) following treatment with antibiotics. There was no difference observed in BMI following patient recovery (mean 20.2 kg/m^2^ [PEx1] vs. 20.6 kg/m^2^ [PEx2]; *p* = 0.242).

**Fig. 3.**
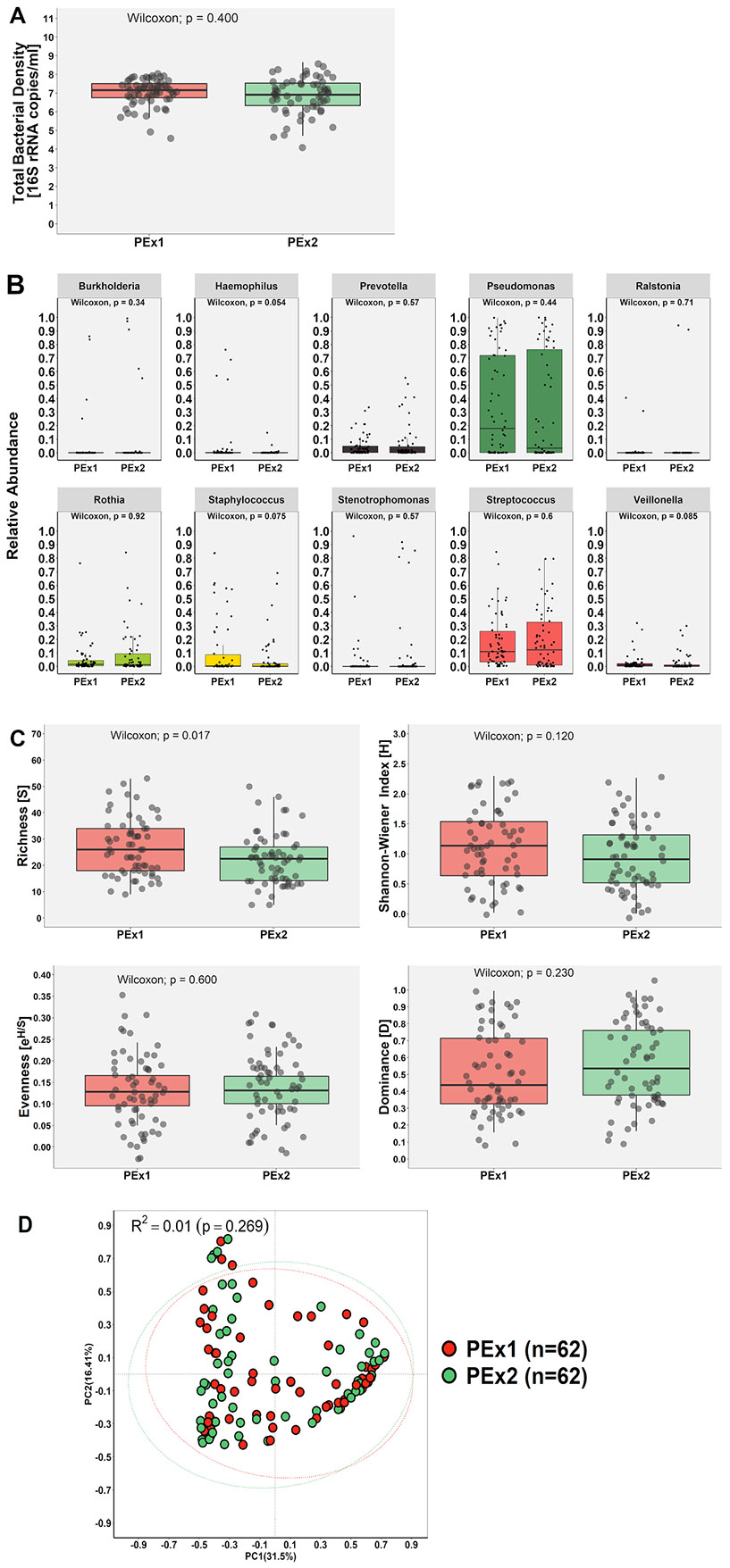
Comparison of the airway microbiota in sputum samples collected at the start (‘PEx1’) and end (‘PEx2’) of antibiotic treatment of a pulmonary exacerbation. **(A**) Total bacterial density quantification by qPCR corresponding to the total number of 16S rRNA encoding gene copies per mL of sputum. **(B)** Relative abundance of top 10 genera detected. **(C)** Ecological parameters, taxonomic richness [S], community diversity (Shannon Wiener Index [H]), evenness [e^H/S^] and dominance [D]. In the box and whisker plots in figures A-C, the line inside the box indicates the median and the top and bottom of the box indicate the 25th and 75th percentile, respectively. The whiskers indicate the 90% CI. Samples were compared using the Wilcoxon-signed rank test **(D)** Principal coordinate analysis (PCoA) plot comparing microbial communities based the adonis analysis with Hellinger transformed data and Bray-Curtis distance (R^2^=0.01; *p* = 0.269, 9999 permutations; confidence based on 90% CI).

There was also no significant difference in bacterial density between sampling timepoints (7.15 [6.75–7.51] copies/mL [PEx1: median; 25th and 75th percentiles; Log10] vs. 6.91 [6.33–7.53] copies/mL [PEx2: median; 25th and 75th percentiles; Log10]; *p* = 0.400; Fig. 3A). Similarly, there was no statistical difference in the relative abundance of the 10 most dominant taxa between timepoints (*p*>0.05) (Fig. 3B). However, the relative abundance of *Haemophilus* spp. (mean proportion: 4.5% [PEx1] vs. 0.4% [PEx2]; *p* = 0.054) and *Staphylococcus* spp. (mean proportion: 11.9% [PEx1] vs. 5.5% [PEx2]; *p* = 0.075) trended towards a significant reduction following antibiotic treatment (Fig. 3B). Moreover, comparison of the occurrence, relative abundance of the taxa or similarity between paired samples demonstrated significant intra- and inter-patient variation (Figs. S6A and S6B, respectively). Community richness [S] was significantly reduced following antibiotic treatment (Fig. 3C; *p* = 0.017) and there was a weak inverse relationship in the difference observed in the within-patient FEV_1_ %predicted and richness [S] (*r*=−0.290; *p* = 0.047) between PEx1 and PEx2. There was no significant difference in Shannon-Wiener Index [H], evenness [e^H/S^] or dominance [D] (Fig. 3C; *p*>0.05). Finally, no difference was observed in community structures between the two sampling time-points (adonis analysis with Hellinger transformed data and Bray-Curtis distance, R^2^=0.01; *p* = 0.269, 9999 permutations) when beta-diversity was compared (Fig. 3D).

Various shifts in the main alpha-diversity indices were observed in response to treatment when samples were compared after stratifying participants by their relative abundance of *Pseudomonas* spp. in the PEx1 sample (relative abundance: ≥75%, *n* = 14; <75%, *n* = 48; Fig. S7). In those with ≥75% relative abundance *Pseudomonas* spp., Shannon-Wiener Index [H] (*p* = 0.024) and evenness [e^H/S^] (*p*<0.001) increased, while dominance [D] decreased (*p* = 0.014) following treatment. In those with <75% relative abundance *Pseudomonas* spp., there was a significant reduction in richness (*p* = 0.015) and Shannon-Wiener Index [H] (*p* = 0.002) and increased dominance [D] (*p* = 0.005) following treatment. A significant difference was also observed in beta-diversity with the relative abundance groups accouting for ~9.1% of the variance explained (adonis analysis with Hellinger transformed data and Bray-Curtis distance, R^2^=0.091; *p* = 0.001, 9999 permutations) (Fig. S7C).

### 3.4. Predicting the likelihood of future exacerbations

To investigate predictors of a pulmonary exacerbation, participants were stratified as cases (*n* = 30) if they had an exacerbation during the study or controls (*n* = 72) if they did not and their baseline stable characteristics were compared.

Cases and controls had a similar baseline age, CFTR function, and comorbidities. Table 2 shows that for the microbiota characteristics analysed, the odds of being a case was significantly reduced with increasing community richness [S] (*p* = 0.038). The odds of being a case was significantly reduced with higher lung function (*p* = 0.001) and increasing BMI (*p* = 0.043). Amongst long-term treatments currently prescribed, azithromycin (*p* = 0.046) and inhaled anti-pseudomonal antibiotics (*p* = 0.001) significantly increased the odds of being a case compared to not being prescribed these treatments. However, the minimum cross-validation criterion using lasso regression resulted in the selection of FEV_1_ %predicted, BMI and inhaled antibiotics as covariates (Table 3). The estimated p-values and confidence intervals suggested that only FEV_1_ %predicted showed a significant effect on the outcome of the model (*p*<0.014; Table 3). The trajectory of cross-validation misclassification rate estimates is shown in the online supplement (Fig. S8). The same outcome was found in the sensitivity analysis using the alternative cut-off periods (online supplement; Table S2).

## 4. Discussion

Whether culture-independent molecular analysis of the lower airway microbiota could be used as an adjunct assessment in the clincal care of PWCF is unclear. Here we provide the results of a study comprised of a prospectively collected dataset with multiple timepoints linking microbial community density and alpha-diversity metrics with clinical outcomes. Although we observed that PWCF harboured their own specific sputum microbiota community, our study clearly demonstrated that the lower airway microbiota had a stable trajectory (i.e. it is conserved) over the time period studied, even with acute IV antibiotic perturbation. Of potential clinical interest, we determined that a shift in microbiota community richness correlated best with patient response to antibiotic treatment of a pulmonary exacerbation. We also observed that various changes occurred in the alpha-diversity metrics, but not the total bacterial density, during these episodes in patients stratified by their *Pseudomonas* relative abundance. This implies that the antibiotic regimens used are having an impact on the sputum community without altering the microbial density. Furthermore, we expected that some microbiota community characteristics such as taxonomic richness might be potentially useful as predictors of a future pulmonary exacerbation in addition to clinical parameters based on the univariate analysis. However, a lasso regression analysis indicated that only the latter, and in particular lower FEV_1_ %predicted, had a greater predictive value in this cohort.

We observed a high degree of inter-patient variability in sputum microbiota community characteristics as previously reported, [4] but the effects of study site were minimal. Our overall finding that the microbiota remained conserved over an 8 month period, and the fact that there was no clear difference in bacterial density, composition or structure between those who received treatment for a pulmonary exacerbation and those who did not, is consistent with a lower airway ecosystem that is resilient and rapidly resets after intense antibiotic pressure [4,10,30]. Although PWCF in our study had moderate to advanced lung disease, no significant change in either microbiota composition or lung function was observed. This confirms results from previous studies which reported that a sustained decrease in CF sputum microbiota diversity occurs over a much longer time frame (~10 years) in those with more severe disease status [4]. However, change in the microbiota over time was observed in a small subset of patients with the highest relative abundance of *Pseudomonas* spp. in their baseline sample; this may reflect the fact that patients chronically colonised with *P. aeruginosa* are more likely to be taking long-term treatments (Fig. S4).

Our understanding of the pathophysiology of infection in CF has been enhanced by culture-independent analysis of the lower airway microbiota, but it has not had a significant impact on clinical practice to date. Often a reduction in the total viable count of recognised pathogens as determined by culture is not observed with antibiotic treatment. Therefore, if molecular analysis identified consistent changes in microbiota metrics, this could potentially be helpful to monitor success of treatment. In addition, we investigated whether there were shifts in sputum microbiota characteristics following use of IV antibiotics that correlated with clinical response to treatment of a pulmonary exacerbation. It is recognised that aggressive antibiotic treatment of these events disturbs the lower airway [4,30,31]. For example, it has been reported that microbiota characteristics such as diversity increase early in treatment (day 3) but then return to pre-treatment levels by days 8–10 [30]. In contrast, in our cohort, during treatment of an exacerbation (mean duration, 17 days), improvement in lung function occurred in parallel with a decrease in community richness. This decrease in richness was also observed in those with a lower, but not higher, relative abundance of *Pseudomonas* spp. Overall, there were no significant changes in the other sputum microbiota characteristics investigated e.g., total bacterial density, dominance and taxa detected and the relative abundance of the top taxa, but various effects were observed when the cohort was split arbitrarily by *Pseudomonas* spp. relative abundance. The data suggest that antibiotics have complex effects on community structure including an effect on other non-target, less common and abundant members of the microbiota. These findings are consistent with a previous study using pyrosequencing to investigate the lower airway microbiota during treatment of exacerbations [32]. Furthermore, a recent study also detected a shift in non-dominant microbiota taxa during inhaled tobramycin suppressive therapy [33].

Whilst others have considered if shifts in the CF lower airway microbiota occur at the onset of a pulmonary exacerbation,[14,34] we investigated whether the sputum microbiota at clinical stability could be used to predict a future exacerbation, similar to our previous study using extended quantitative culture data but in a larger cohort [10]. If the microbiota could be used as a surrogate marker to help predict aspects of clinical course is currently of great interest. One study found that a low sputum microbiota diversity could predict early disease progression [7] and another reported that a higher abundance of *Staphylococcus* before treatment with long-term inhaled tobramycin was potentially related to patient response [17]. In the present study, there was no microbiota metric that associated with a future exacerbation based on the lasso regression model. However, lower lung function during clinical stability was a predictor of an exacerbation (univariate analysis), which corroborates the findings of others [35].

Limitations of our study were, firstly, that not all study participants were included in the analyses due to missing samples at target timepoints when they were unable to expectorate any or only a small volume of sputum, as previously reported [10]. Secondly, the type of analyses used here does not account for the complex interplay of microbe–microbe and microbe–host interactions that might occur in vivo. Therefore, further studies could investigate, for example, the metagenome, metatranscriptome and metabolome to begin to unravel the dynamic functional differences that occur in the lower airways longitudinally [33,36,37]. Thirdly, our study was exploratory and as analyses of the sputum microbiota requires considerable expense and expertise, larger prospective clinical trials would be required to assess the feasibility and precision of using the microbiota as an adjunct assessment to clinical investigations across various age groups and stages of disease. Furthermore, whether microbiota associated metrics could be used to help inform selection of antibiotics, guide duration of therapy or identify those who do not respond to treatments are of interest and require further investigation [17,30,38,39]. Refinement of measures such as improved read resolution for species identification or only recruiting participants with chronic *P. aeruginosa* infection may reveal other associations with clinical outcomes. Moreover, in the era of CFTR modulators, where the collection of sputum is more challenging it may not be practical to implement in clinical practice [40].

## 5. Conclusion

In this longitudinal prospective study, we show overall that PWCF harbour a lower airway microbiota with a stable trajectory over an 8 month period. Our findings demonstrate that a shift in sputum microbiota richness occurred simultaneously with treatment response of a pulmonary exacerbation. We also observed some differences in the baseline sputum microbiota between PWCF who had an exacerbation and those who remained clinically stable, but these differences did not predict a future pulmonary exacerbation in our final statistical model. Nevertheless, sputum diversity metrics may provide additional insights to patient-level measurements, including to help monitor response to antibiotics.

## Supplementary Material

Supplemental

Figure S8

Figure S2

Figure S1

Figure S3

Figure S4

Figure S7

Figure S5

Figure S6

Supplementary material associated with this article can be found, in the online version, at doi:10.1016/j.jcf.2023.11.010.

## Figures and Tables

**Table 1:** Participant demographics and clinical information collected at study enrolment.

	Study site* (number of participants^†^)				
	All (*n* = 149)	Belfast (*n* = 85)	Dublin (*n* = 20)	Chapel Hill (*n* = 44)	p-value^‡^
Age (years); mean (SD)	26.3 (12.2)	30.5 (13.7)	23.1 (7.2)	19.8 (6.5)	<0.001
Female gender; n (%)	68 (44.6)	35 (41.2)	10 (50.0)	23 (52.3)	0.4
***CFTR function; n (%)***					na
Minimal	109 (73.2)	54 (63.5)	18 (90.0)	37 (84.1)	
Residual	16 (10.7)	16 (18.8)	0 (0)	0 (0)	
Non-classified	24 (16.1)	15 (17.6)	2 (20.0)	7 (15.9)	
***Clinical measurements; mean (SD)***					
FEV_1_ %predicted; mean (SD)	62.1 (12.2)	65.8 (22.3)	46.8 (19.2)	61.7 (22.9)	0.004
BMI (kg/m^2^); mean (SD)	21.7 (3.89)	22.6 (4.1)	19.7 (2.9)	20.4 (3.0)	<0.001
***Co-morbidities; n (%)***					
Pancreatic insufficient	136 (91.3)	77 (90.1)	20 (100)	39 (88.6)	na
CF-related diabetes	27 (19.4)	10 (11.8)	10 (50.0)	7 (20.6)	0.001
Liver disease	11 (8.0)	1 (1.2)	3 (15.0)	7 (21.2)	na
***Chronic oral treatments; n (%)***					
Flucloxacillin	8 (5.4)	8 (9.4)	0 (0)	0 (0)	na
Azithromycin	103 (69.6)	55 (64.7)	13 (68.4)	35 (79.5)	0.2
***Chronic inhaled antibiotics; n (%)***					
Tobramycin	54 (36.5)	26 (30.6)	9 (47.4)	19 (43.2)	0.2
Colistin	28 (18.9)	25 (29.4)	4 (21.1)	0 (0)	0.001
Aztreonam	6 (4.1)	0 (0)	0 (0)	6 (13.6)	na
Other	4 (2.7)	1 (1.2)	0 (0)	3 (6.8)	na
None	55 (37.2)	33 (38.8)	6 (31.6)	16 (36.4)	0.8
***Other treatments; n (%)***					
DNase	116 (78.4)	64 (75.3)	14 (73.7)	38 (86.4)	0.3
Hypertonic saline	52 (35.1)	14 (16.5)	12 (63.2)	26 (59.1)	<0.001
Antacid	74 (48.3)	33 (38.8)	14 (73.6)	27 (62.8)	0.03
Insulin	23 (15.6)	7 (8.2)	8 (42.1)	8 (18.6)	0.001

Definitions: SD, standard deviation; CFTR, cystic fibrosis transmembrane conductance regulator (function classed as follows: minimal, harbouring two alleles with Class I-III mutations; residual, harbouring ≥1 allele with Class IV-V mutations; non-classified, not enough information available to class function as residual or minimal); FEV_1_ %predicted, forced expiratory volume in one-second%predicted; BMI, body mass index; NA, non-applicable when expected counts were less than 5 for >20% of the cells.

* CF centres in Belfast in the UK (Adult and Paediatric CF Centres, Belfast Health and Social Care Trust), Dublin in Ireland (Beaumont Hospital and Our Lady’s Children’s Hospital, Crumlin) and University of North Carolina (UNC) at Chapel Hill, NC in the USA (UNC Hospital Pediatric and Adult Clinics).

† Not all participants had data available for all variables: FEV_1_ %predicted measurement and CF-related diabetes status, *n* = 139; liver disease status, *n* = 138; chronic treatments, inhaled antibiotics, DNase, and hypertonic saline prescribed, *n* = 148; antacid and insulin prescribed, *n* = 147.

‡ The p-value presented relates to the comparison between the three CF centres.

**Table 2:** Baseline stable clinical and sputum community characteristics and their association with being a case.

Co-variates	Cases (*n* = 30)	Controls (*n* = 72)	OR (95% CI)^†^	p-value
**Clinical parameters**				
Age (years); mean (SD)	27.0 (12.7)	27.9 (13.8)	na	na
Female gender; n (%)	18 (60.0)	29 (40.3)	na	na
*Study site; n (%)*				
Belfast	21 (70.0)	45 (62.5)	na	na
Dublin	3 (10.0)	3 (4.2)	na	na
Chapel Hill	6 (20.0)	24 (33.3)	na	na
FEV_1_ %predicted; mean (SD)*	53.9 (21.9)	71.5 (18.8)	0.949 (0.921–0.978)	0.001
BMI (kg/m^2^); mean (SD)	21.2 (3.0)	23.0 (4.3)	0.850 (0.725–0.995)	0.043
Pancreatic insufficient; n (%)*	27 (90.0)	62 (86.1)	1.176 (0.276–5.021)	0.827
CF-related diabetes; n (%)*	3 (10.3)	9 (13.0)	0.522 (0.113–2.400)	0.403
Liver disease; n (%)*	1 (3.5)	7 (10.1)	0.236 (0.021–2.709)	0.246
*CFTR function; n (%)*				
Minimal	24 (80.0)	46 (63.9)	3.161 (0.777–12.859)	0.108
Residual	3 (10.0)	11 (15.3)	1.684 (0.255–11.101)	0.588
Non-classified	3 (10.0)	15 (20.8)	Reference	
*Chronic treatments; n (%)*				
Oral azithromycin	23 (76.7)	45 (62.5)	2.987 (1.021–8.735)	0.046
Oral flucloxacillin	1 (3.3)	3 (4.2)	0.666 (0.058–7.690)	0.745
Inhaled antibiotics	25 (88.3)	33 (45.8)	5.981 (1.983–18.041)	0.001
**Sputum community characteristics**				
Richness [S]; mean (SD)	29.9 (8.9)	33.1 (9.3)	0.946 (0.897–0.997)	0.038
Shannon-Wiener Index [H]; mean (SD)	1.3 (0.5)	1.2 (0.5)	1.194 (0.461–3.091)	0.715
Evenness [e^H/S^]; mean (SD)	0.1 (0.0)	0.1 (0.1)	1.103 (0.998–1.219)^‡^	0.054
Dominance [D; mean (SD)	0.5 (0.2)	0.5 (0.2)	0.394 (0.040–3.860)	0.424
Log10 total 16S rRNA copy number per mL; median (25th percentile and 75th percentile)*	7.3 (6.8–7.6)	7.3 (6.9–7.7)	0.963 (0.570–1.625)	0.887
*Pseudomonas* dominance (≥75% relative abundance), n (%)	3 (10.0)	11 (18.0)	0.696 (0.168–2.857)	0.613
*Relative abundance of top 10 taxa (%); mean (SD)*				
*Streptococcus*	27.4 (19.2)	31.8 (23.3)	0.988 (0.967–1.010)	0.281
*Pseudomonas*	25.4 (32.3)	25.5 (34.9)	1.000 (0.987–1.014)	0.959
*Staphylococcus*	8.6 (19.0)	9.7 (21.8)	0.995 (0.973–1.019)	0.698
*Prevotella*	6.5 (10.3)	7.6 (10.2)	0.988 (0.941–1.037)	0.618
*Rothia*	5.3 (8.8)	3.9 (5.9)	1.034 (0.974–1.099)	0.274
*Haemophilus*	3.7 (12.9)	4.2 (16.0)	0.994 (0.963–1.025)	0.684
*Burkholderia*	4.1 (12.9)	3.6 (13.4)	1.010 (0.977–1.043)	0.573
*Achromobacter*	4.5 (18.3)	2.1 (9.9)	1.016 (0.982–1.051)	0.356
*Stenotrophomonas*	5.1 (16.1)	0.3 (2.1)	1.160 (0.980–1.374)	0.084
*Veillonella*	1.7 (2.3)	3.0 (3.9)	0.859 (0.707–1.044)	0.128

† Odds of being a case compared to being a control are provided. All odds ratios presented are adjusted for age, gender and site due to their expected effects on odds of being diagnosed with an exacerbation; only gender was statistically significant in some of the models.

II Continuous variables were analysed per unit increase.

* Missing data as follows: FEV_1_ %predicted, cases *n* = 10 and controls, *n* = 8; CF-related diabetes and liver disease, cases *n* = 1 and controls, *n* = 3; Log10 total 16S rRNA (copy number per mL), cases, *n* = 1.

‡ Values transformed via multiplying by the factor of 100 before inclusion in the logistic regression model.

Definitions: SD, standard deviation; FEV_1_ %predicted, forced expiratory volume in one-second%predicted; BMI, body mass index; CFTR, cystic fibrosis transmembrane conductance regulator (function classed as follows: minimal, harbouring two alleles with Class I-III mutations; residual, harbouring ≥1 allele with Class IV-V mutations; non-classified, not enough information available to class function as residual or minimal); OR, odds ratio; CI, confidence interval.

**Table 3:** Results from the lasso regression on the outcome of a future exacerbation.

Co-variates	Coefficient	Z-score	p-value	Lower CI	Upper CI	Lower tail area	Upper tail area
FEV_1_ %predicted	0.710	2.601	0.014	0.281	3.561	0.049	0.050
BMI	0.289	1.045	0.926	−16.433	0.189	0.050	0.049
Inhaled antibiotics	0.463	1.723	0.079	−0.184	4.121	0.050	0.050

*Definitions:* FEV_1_ %predicted, forced expiratory volume in one-second%predicted; BMI, body mass index, CI, confidence interval.

## Data Availability

All data relevant to the study are included in the article or uploaded as supplementary information. The accession number for all amplicon sequencing data reported in this paper can be found at NCBI-SRA under Bioproject Accession Number PRJNA865935.
