# Supplementary material for: Longitudinal changes in the cystic fibrosis airway microbiota with time and treatment
Source: J Cyst Fibros. Author manuscript; Available in PMC 2026 Jan 23. (PMC12829167; doi:10.1016/j.jcf.2023.11.010)
Supplement: Supplemental [file NIHMS2131163-supplement-Supplemental.docx]

**Online Supplement**

### Title Longitudinal changes in the cystic fibrosis airway microbiota with time and treatment

Einarsson, Gisli G.^1*^, Sherrard, Laura J.^1*^, Hatch, Joseph E.^3^, Zorn, Bryan^3^, Johnston, Elinor^1^, McGettigan, Clodagh^1^, O’Neill, Katherine.^1^, Gilpin, Deirdre F.^1^, Murray, Michelle^2^, Lavelle, Gillian^2^, McElvaney, Gerry^2^, Wolfgang, Matthew C.^3^, Boucher, Richard^3^, Muhlebach, Marianne S.^3^, Bradbury, Ian^4^, Elborn, J. Stuart^1‡^, Tunney, Michael M.^1‡^

^1^ Queen’s University Belfast, Belfast, United Kingdom.

^2^ Royal College of Surgeons in Ireland, Beaumont Hospital, Dublin, Ireland

^3^ University of North Carolina, Chapel Hill, North Carolina, United States of America

^4^ Frontier Science (Scotland) Ltd., Kincraig, United Kingdom

* GGE and LJS are joint first authors on this paper

^‡^ JSE and MMT are joint senior authors on this paper

**Corresponding author** Gisli G. Einarsson Queen’s University Belfast, Belfast, United Kingdom. Email: [g.einarsson@qub.ac.uk](mailto:g.einarsson@qub.ac.uk)

**METHODS**

**Illumina MiSeq sequencing and data pre-processing**

Preparation of samples for Illumina MiSeq sequencing; quality assessment of sample DNA, library preparation, sample pooling and sequencing was performed at the University of North Carolina Chapel Hill as previously described.[1]

**Illumina MiSeq sequencing and data post-processing**

Following the sequencing of the 16S rRNA marker-gene on the Illumina MiSeq platform, quality assessment of raw amplicon reads as well as the detection of potential sequence read contaminants (found in the negative controls) that may have been introduced during the library preparation were processed and removed as previously described.[1] In brief, paired-end Illumina MiSeq sequences were processed using QIIME (Quantitative Insights into Microbial Ecology; version 1.9.1).[2] Following joining of the combined forward and reverse reads using fastq-join (with default settings), sequences were demultiplexed using an in-house AWK script that removed inline primer sequences and retained 16S rRNA amplicon sequences with greater than 85% of bases exceeding a Q-Score of ≥28. Chimeric sequences were detected and removed from the data-set using ChimeraSlayer within QIIME. Sequences were clustered into their representative operational taxonomic units (OTUs) based on 97% sequence identity via the UCLUST algorithm.[3] Sequences were then aligned against full length 16S rRNA marker gene sequences from the GreenGenes reference database (version 13.8)[4] via PyNAST[5] and assigned taxonomic identities according to the Ribosomal Database Project Classifier Tool (version 2.2)[6] using open-reference OTU picking implemented within QIIME. Positive DNA extraction controls (four per 96 well plate; 8 plates in total) were prepared using excess sputum samples and used to monitor inter- and intra-run variation. For the positive controls, the average number of amplicon reads per sample was 38632 (range: 1075-123822). Negative (blank) extraction controls (four per 96 well plate; 8 plates in total) were utilized to monitor potential technical contamination or background signal from extraction and sequencing reagents. To assess the putative effect negative controls might have on the interpretation of the data, the resulting OTU-table from the taxonomic assignment was log-normalized (0 sequence values were converted to 1) and each of the OTUs from the corresponding negative control sample was correlated against the OTUs from the sample wells immediately surrounding it. The average amplicon count per negative control was 4892 (range: 34-23964). The negative from each plate that had the lowest average correlation was assumed to be the negative control sample that was least representative of cross contamination. For each plate, negative extraction controls were included. Negative controls were correlated against one another and each that had any high correlating values (0.50 and above) were selected to determine commonalities among them as a whole. Ranking the averages from high to low resulted in a list of 22 OTUs (11 corresponding taxa), with a summary of OTUs determined as putative contaminants shown in Table S1. These taxa/OTUs sequences were subtracted from the non-normalized dataset to decrease the influence of reagent or processing contamination. Sequences were then rarefied to a common depth of 10000 reads per sample.

**Statistical analysis and microbiota community measurements**

Assessment of the microbiota community data demonstrated that for most of the included variables, the data did not conform to a normal distribution (Shapiro-Wilk normality test; p≤0.05). Data was analysed in a number of different ways. The main focus was comparing the First and Last stable samples and secondly, samples collected at the start of pulmonary exacerbation (PEx1) and at the completion of antibiotic treatment for a pulmonary exacerbation (PEx2). Further exploratory investigations were then performed to assess if occurrence of a pulmonary exacerbation in between First and Last stable samples affected the microbial community composition (exacerbated, n=40; remained stable, n=74). Similarly, further stratification of the participants into tertiles (<25 weeks, n=35; 25-38 weeks, n=40; >39 weeks, n=39) based on time between stable sample collection was undertaken to assess if the number of weeks had an impact on the microbial community composition. Finally, we assessed if community dominance by members of *Pseudomonas* spp. affected factors related to community composition by segregating the participants into groups depending on their relative abundance of the in the First stable sample or the PEx1 sample (≥75% or <75% relative abundance) as previously described.[7] Taxa abundances are shown as relative abundance for normalised amplicon read count data following pre-processing and quality checking of both sample reads and negative controls (normalised to 10000 reads across all samples). Alpha-diversity (within group) indices, taxonomic richness [S], diversity (Shannon-Wiener Index [H]), evenness [e^H/S^] and dominance [D], were calculated in PAST version 3.20 (<http://folk.uio.no/ohammer/past>) and compared between groups using the Wilcoxon-signed-rank test (2 groups). Beta-diversity (between analytical groups) was assessed using distance-based metric (Bray-Curtis distance) on Hellinger transformed OTU table and presented as a principle coordinates plot (PCoA) showing variance explained for the first two components. Differences between groups were evaluated by multivariate-permutational analysis (PERMANOVA) as implemented within the adonis test with 9999 permutations. A canonical analysis of principal coordinates on centred log-ratio (clr)-transformed data was performed followed by the addition of selected variables as constraints to assess their effect on the community structure. Differences between categorical groups were evaluated by PERMANOVA as previously described. The analysis of microbial community based and clinical data was conducted in R version 4.1.2 (R Core Team (2021). R: A language and environment for statistical computing. R Foundation for Statistical Computing, Vienna, Austria. <https://www.r-project.org/>).

To determine factors which could potentially be used to predict a future pulmonary exacerbation, participants were categorised as either cases (diagnosed with an exacerbation within 4 months of a previous stable sample; n=30) or controls (no evidence of an exacerbation during the study and time between study visits was >4 months; n=72) and baseline stable sputum community characteristics and clinical parameters were described using descriptive statistics with adjusted odds (variables expected to affect risk - age, gender, study site - were retained to control for any potential confounding effects) and 95% confidence intervals reported. A lasso regression was used to assess the impact of the covariates on the outcome of a future exacerbation. The continuous variables were standardised, based on an analysis of the misclassification rate, and a 10-fold cross-validation (CV) was used to estimate the rate. Selective inference was used to estimate p-values and confidence intervals correcting for the selection procedure in the process.

**REFERENCES**

[1] Muhlebach MS, Zorn BT, Esther CR, Hatch JE, Murray CP, Turkovic L, et al. Initial acquisition and succession of the cystic fibrosis lung microbiome is associated with disease progression in infants and preschool children. PLoS Pathogens. 2018;14:e1006798.

[2] Caporaso JG, Kuczynski J, Stombaugh J, Bittinger K, Bushman FD, Costello EK, et al. QIIME allows analysis of high-throughput community sequencing data. Nat Methods. 2010;7:335-6.

[3] Edgar RC. Search and clustering orders of magnitude faster than BLAST. Bioinformatics. 2010;26:2460-1.

[4] DeSantis TZ, Hugenholtz P, Larsen N, Rojas M, Brodie EL, Keller K, et al. Greengenes, a chimera-checked 16S rRNA gene database and workbench compatible with ARB. Appl Environ Microbiol. 2006;72:5069-72.

[5] Caporaso JG, Bittinger K, Bushman FD, DeSantis TZ, Andersen GL, Knight R. PyNAST: a flexible tool for aligning sequences to a template alignment. Bioinformatics. 2010;26:266-7.

[6] Wang Q, Garrity GM, Tiedje JM, Cole JR. Naive Bayesian classifier for rapid assignment of rRNA sequences into the new bacterial taxonomy. Appl Environ Microbiol. 2007;73:5261-7.

[7] Acosta N, Heirali A, Somayaji R, Surette MG, Workentine ML, Sibley CD, et al. Sputum microbiota is predictive of long-term clinical outcomes in young adults with cystic fibrosis. Thorax. 2018;73:1016-25.

**Table S1** OTUs (22 OTUs; 10 corresponding taxa) determined as putative contaminants originating from negative controls.

| **OTUs** | **Taxa** |
| --- | --- |
| 720353 | family Comamonadaceae Unclassified |
| 676066 | family Bradyrhizobiaceae Unclassified |
| 1105814 | genus Bradyrhizobium Unclassified |
| 580625 | genus Bradyrhizobium Unclassified |
| 807415 | genus Rhodobacter Unclassified |
| 746799 | genus Rhodobacter Unclassified |
| 1108960 | genus Sphingomonas Unclassified |
| 1003206 | genus Sphingomonas Unclassified |
| 209595 | genus Sphingomonas Unclassified |
| 334067 | family Comamonadaceae Unclassified |
| 783719 | family Comamonadaceae Unclassified |
| 719367 | family Comamonadaceae Unclassified |
| 1106324 | family Comamonadaceae Unclassified |
| 114922 | family Comamonadaceae Unclassified |
| 1111342 | family Oxalobacteraceae Unclassified |
| 802011 | family Methylophilaceae Unclassified |
| 1105514 | family Rhodocyclaceae Unclassified |
| New.ReferenceOTU1889 | family Rhodocyclaceae Unclassified |
| 584324 | family Xanthomonadaceae Unclassified |
| 707290 | family Xanthomonadaceae Unclassified |
| 222534 | family Xanthomonadaceae Unclassified |
| 81358 | family Xanthomonadaceae Unclassified |

**Table S2** Sensitivity analysis

| **FEV_1_ %predicted** | **Coefficient** | **p-value** | **Lower CI** | **Upper CI** |
| --- | --- | --- | --- | --- |
| 3 months cut-off period | 0.72 | 0.08 | -0.14 | 1.81 |
| 6 months cut-off period | 0.57 | 0.06 | -0.06 | 1.02 |

*Definitions:* FEV_1_ %predicted, forced expiratory volume in one-second %predicted; CI, confidence interval.

**FIGURE LEGENDS**

**Figure S1** Comparison of microbial community composition in paired CF (n=114) samples at ‘First’ and ‘Last’ stable time-points. Relative abundance shown for the top 15 genera.

**Figure S2** Comparison of sputum microbiota characteristics at clinical stability between participants when stratified based on their pulmonary exacerbation status (remained stable, ‘Stable Group’, n=74; exacerbated, ‘PEx Group’, n=40) in between collection of the ‘First’ and ‘Last’ stable samples. **(A**) Total bacterial load quantification by qPCR corresponding to the total number of 16S rRNA encoding gene copies per mL of sputum. **(B)** Ecological parameters, taxonomic richness [S], community diversity (Shannon-Wiener Index [H]), evenness [e^H/S^] and dominance [D]. In the box and whisker plots (figures A and B), the line inside the box indicates the median and the top and bottom of the box indicate the 25^th^ and 75^th^ percentile, respectively. The whiskers indicate the 90% confidence interval (CI). Samples were compared using a Wilcoxon-signed rank test **(C)** Principal coordinate analysis (PCoA) plot comparing microbial communities based on adonis analysis with Hellinger transformed data and Bray-Curtis distance (R^2^=0.01; p=0.034; 9999 permutations; confidence based on 90% CI).

**Figure S3** Comparison of sputum microbiota characteristics at clinical stability between participants when stratified based on the duration of time (<25 weeks, n=35; 25-38 weeks, n=40; >39 weeks, n=39) between collection of their ‘First’ and ‘Last’ stable samples. **(A**) Total bacterial load quantification by qPCR corresponding to the total number of 16S rRNA encoding gene copies per mL of sputum. **(B)** Ecological parameters, taxonomic richness [S], community diversity (Shannon-Wiener Index [H]), evenness [e^H/S^] and dominance [D]. In the box and whisker plots (figures A and B), the line inside the box indicates the median and the top and bottom of the box indicate the 25^th^ and 75^th^ percentile, respectively. The whiskers indicate the 90% confidence interval (CI). Samples were compared using a Wilcoxon-signed rank test **(C)** Principal coordinate analysis (PCoA) plot comparing microbial communities (adonis analysis with Hellinger transformed data and Bray-Curtis distance R^2^=0.01; p=0.034; 9999 permutations; confidence based on 90% CI).

**Figure S4** Canonical analysis of principal coordinates (CAP) on clr-transformed data. The CAP-biplot shows the loading of each selected variable (arrows) and the scores of each sample (points). The length of the arrows approximates the variance of the variables, whereas the angles between them approximate the correlation between them. Points close together correspond to observations that have similar PCoA component scores. The ellipses show the 90% confidence interval scores for each site. The effect of study site on interpretation of the data were evaluated by multivariate-permutational analysis (PERMANOVA) as implemented within the adonis test with 9999 permutations, with sampling site accounting for 2.7% of the explained variance (p=0.002).

**Figure S5** Comparison of sputum microbiota characteristics at clinical stability between participants when stratified based on *Pseudomonas* spp. relative abundance (≥75%, n=15; <75%, n=99) in their ‘First’ stable sample. **(A**) Total bacterial load quantification by qPCR corresponding to the total number of 16S rRNA encoding gene copies per mL of sputum. **(B)** Ecological parameters, taxonomic richness [S], community diversity (Shannon-Wiener Index [H]), evenness [e^H/S^] and dominance [D]. In the box and whisker plots (figures A and B), the line inside the box indicates the median and the top and bottom of the box indicate the 25^th^ and 75^th^ percentile, respectively. The whiskers indicate the 90% confidence interval (CI). Samples were compared using a Wilcoxon-signed rank test. **(C)** Principal coordinate analysis (PCoA) plot comparing microbial communities (adonis analysis with Hellinger transformed data and Bray-Curtis distance R^2^=0.011; p=0.044; 9999 permutations; confidence based on 90% CI).

**Figure S6** Comparison of microbial community composition in paired (n=62) samples at the start of (‘PEx1’) and completion of (‘PEx2’) antibiotic treatment of a pulmonary exacerbation. **(A)** Relative abundance shown for the top 15 genera. **(B)** Hierarchical cluster dendrogram showing sample-wise similarity between paired samples (Bray–Curtis dissimilarity based on the variance criterion of the WARD.D2 cluster method). Tips of the dendrogram denote matching of sample pairs as highly similar (blue; sample pair on a same minor branch); somewhat similar (green; sample pairs of closely positioned minor branches) and dissimilar (red; sample pair separated onto different major branches.

**Figure S7** Comparison of the airway microbiota in sputum samples collected at the start (‘PEx1’) and end (‘PEx2’) of antibiotic treatment of a pulmonary exacerbation between participants stratified based on *Pseudomonas* spp. relative abundance (≥75%, n=14; <75%, n=48) in their ‘PEx1’ sample. **(A**) Total bacterial load quantification by qPCR corresponding to the total number of 16S rRNA encoding gene copies per mL of sputum. **(B)** Ecological parameters, taxonomic richness [S], community diversity (Shannon-Wiener Index [H]), evenness [e^H/S^] and dominance [D]. In the box and whisker plots (figures A and B), the line inside the box indicates the median and the top and bottom of the box indicate the 25^th^ and 75^th^ percentile, respectively. The whiskers indicate the 90% confidence interval (CI). Samples were compared using a Wilcoxon-signed rank test **(C)** Principal coordinate analysis (PCoA) plot comparing microbial communities based on adonis analysis with Hellinger transformed data and Bray-Curtis distance (R^2^=0.091; p=0.001, 9999 permutations; confidence based on 90% CI).

**Figure S8** Trajectory of CV Misclassification Rate Estimates for lasso regression*.* In our estimation, the null model without covariates gave a misclassification rate of 29.67% and the final estimated misclassification rate from the selected model was 25.27%.
