# Supplementary figures and images for: Longitudinal changes in the cystic fibrosis airway microbiota with time and treatment
Source: J Cyst Fibros. Author manuscript; Available in PMC 2026 Jan 23. (PMC12829167; doi:10.1016/j.jcf.2023.11.010)

Misclassification Error

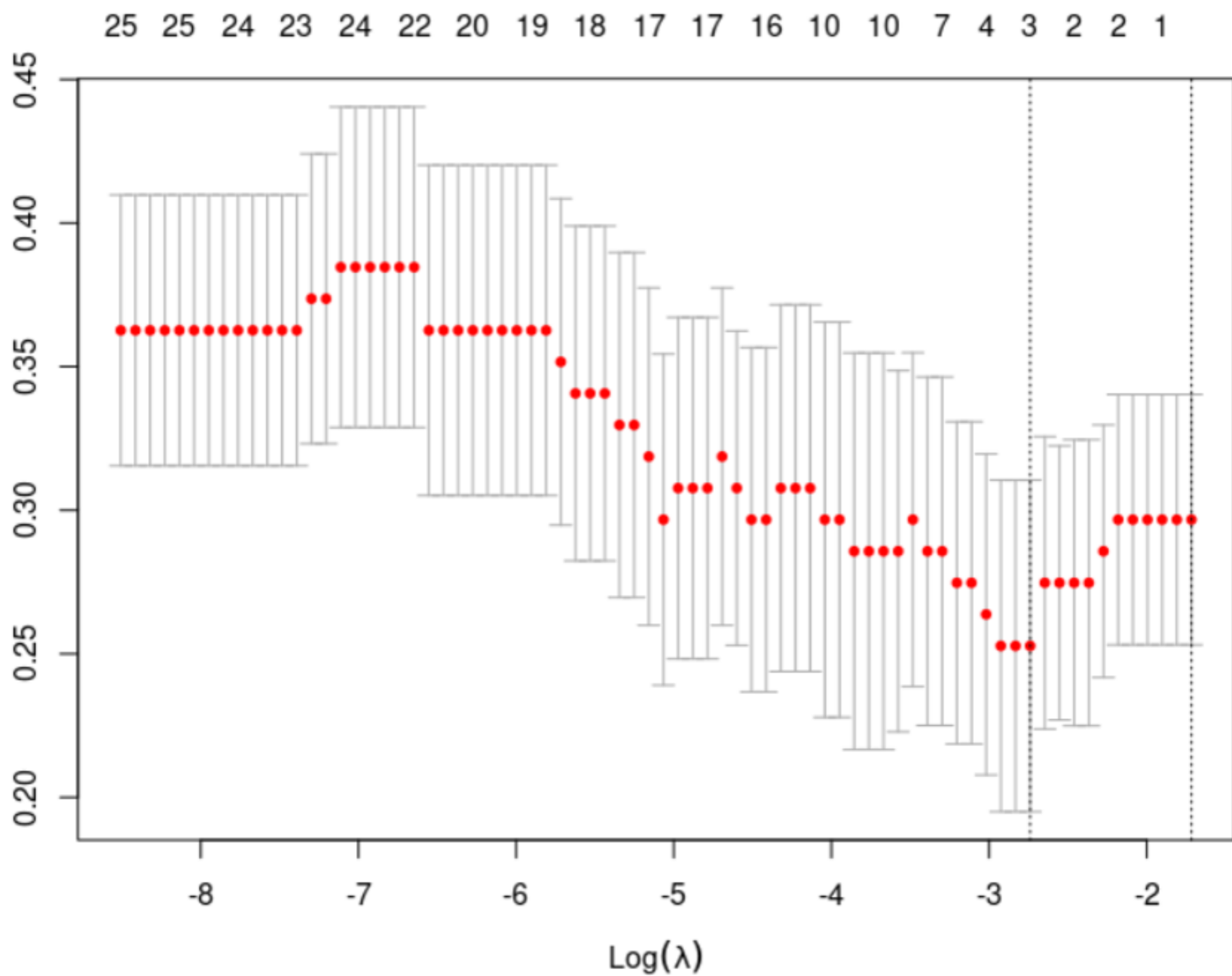

Supplement: Figure S8 [file NIHMS2131163-supplement-Figure_S8.pdf]

**A**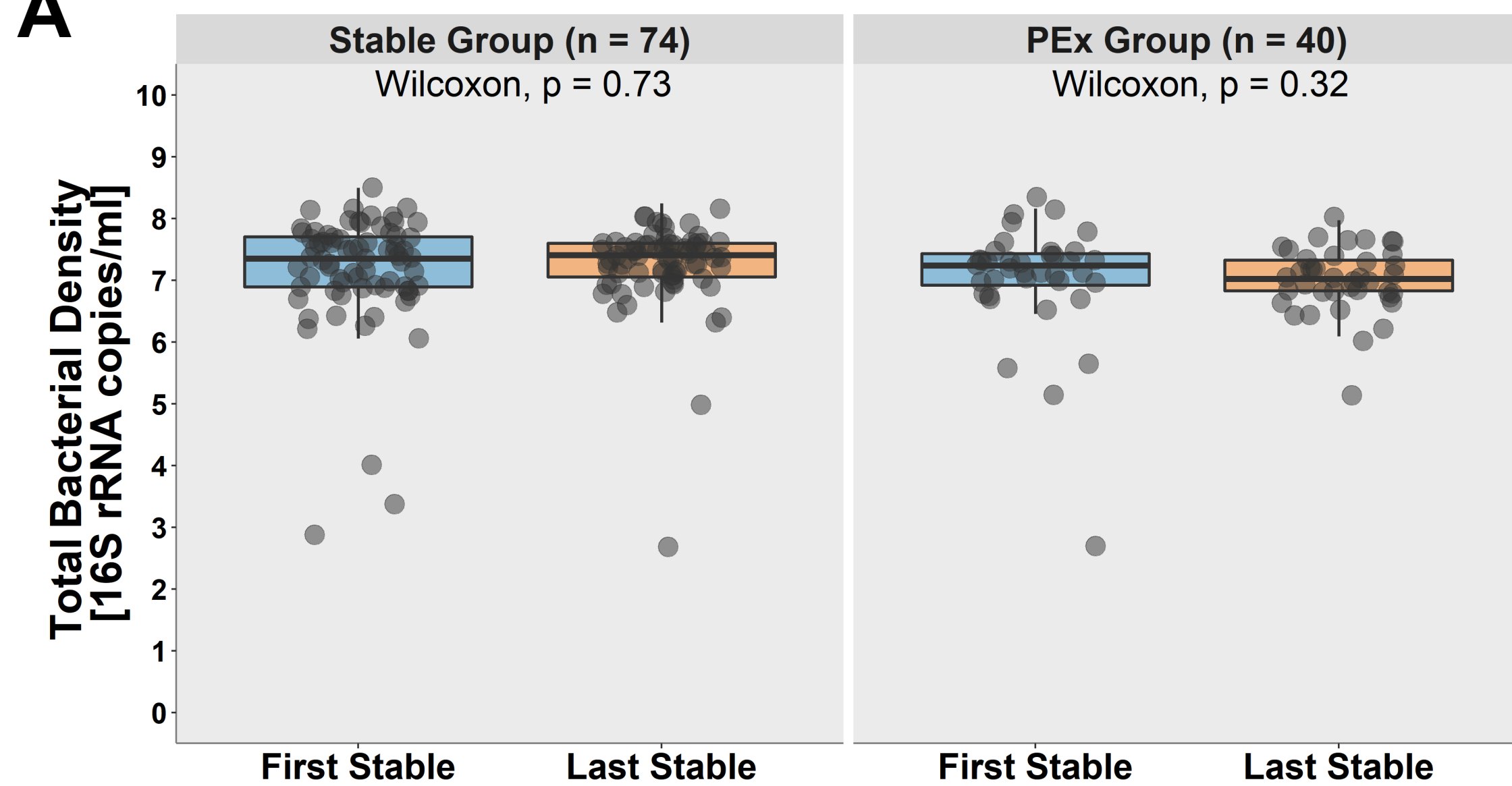**B**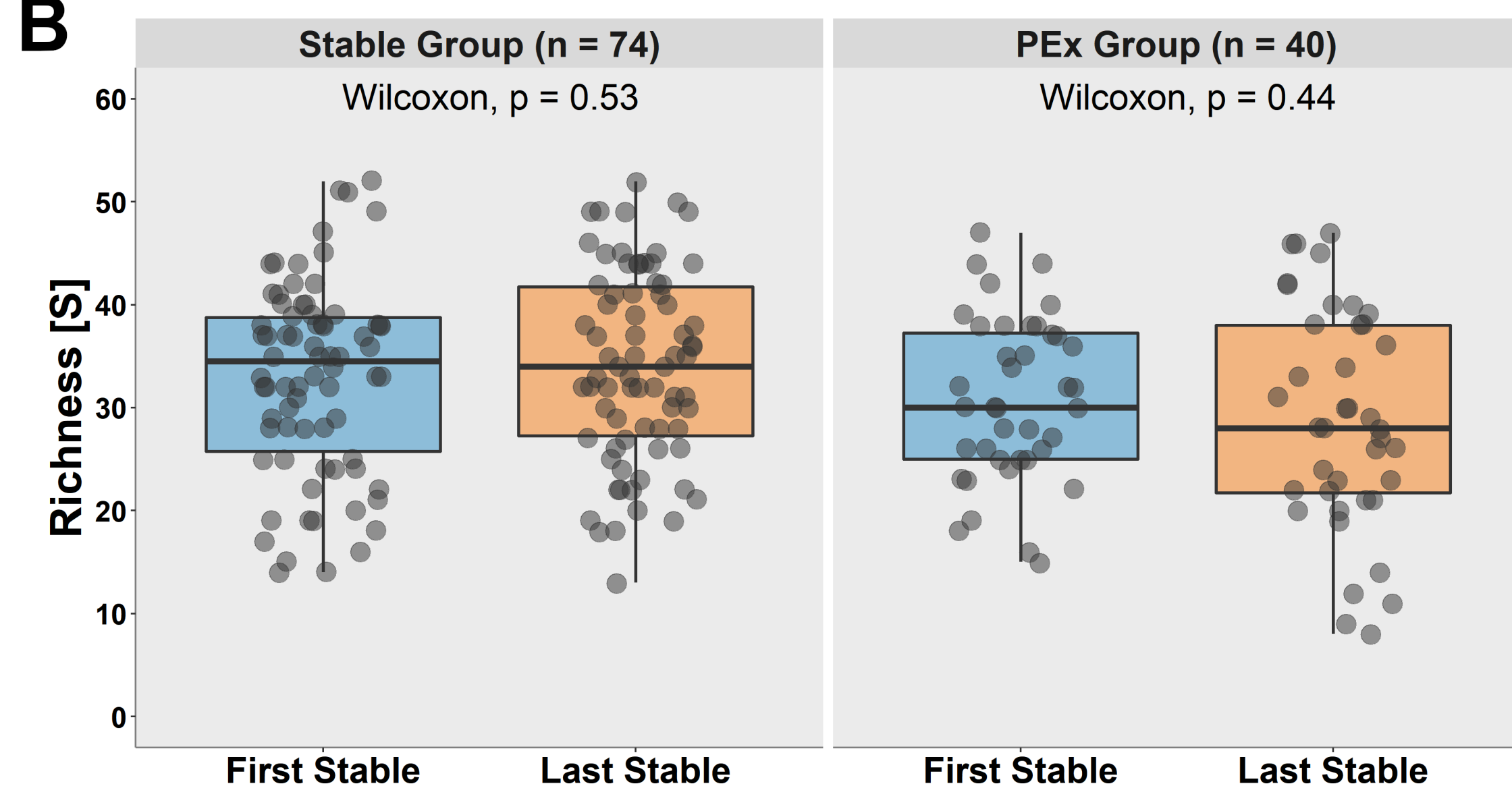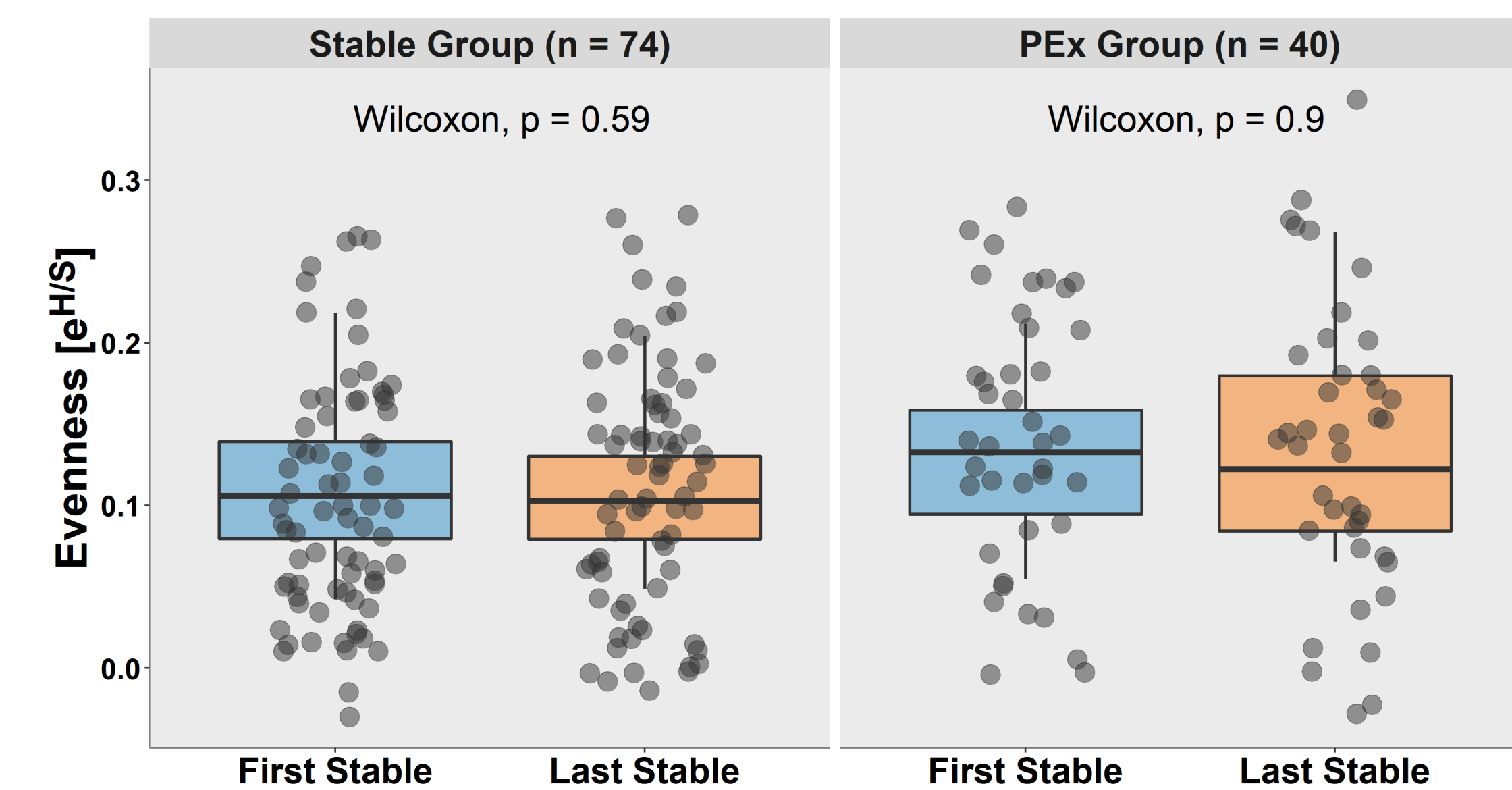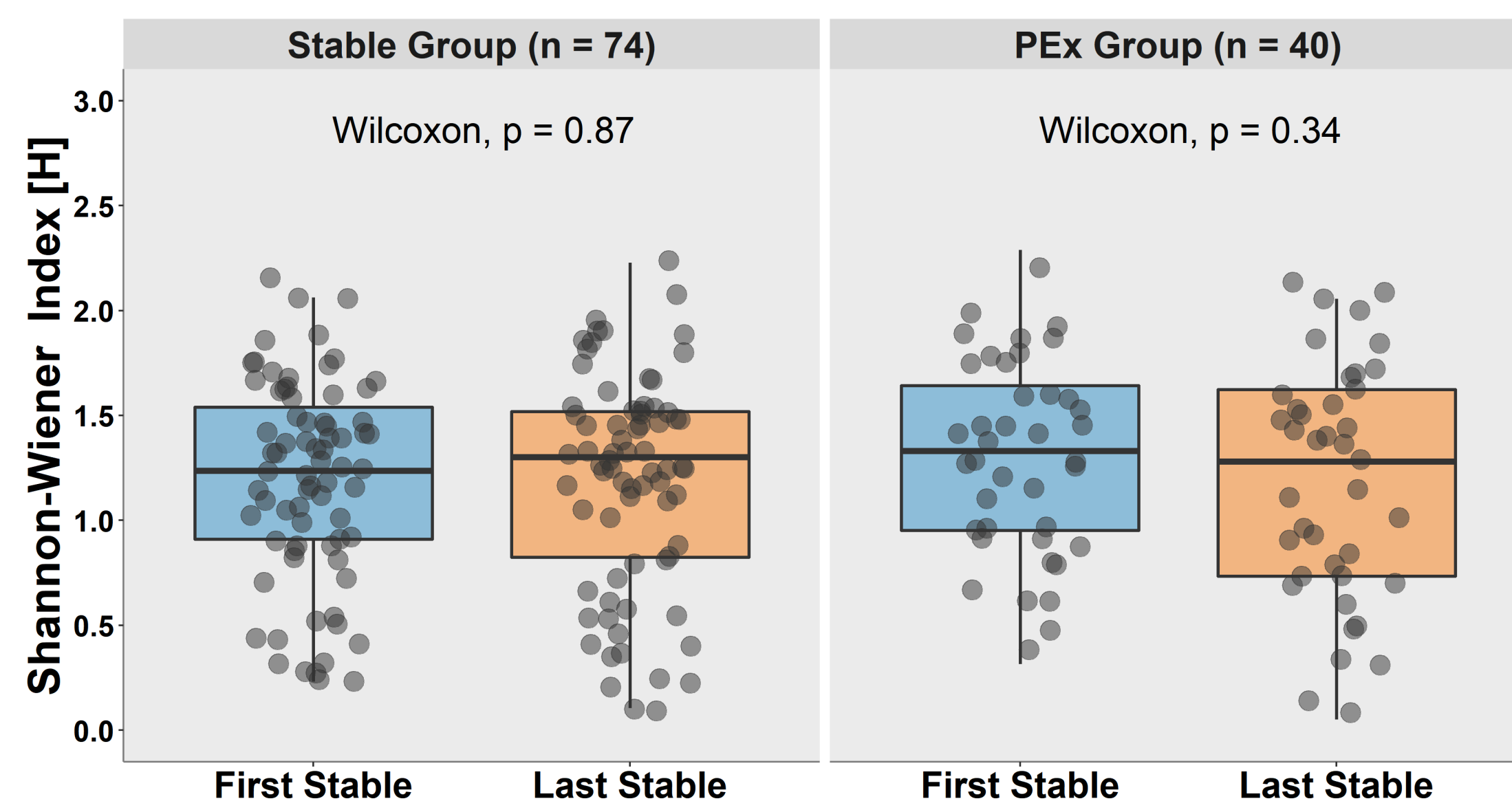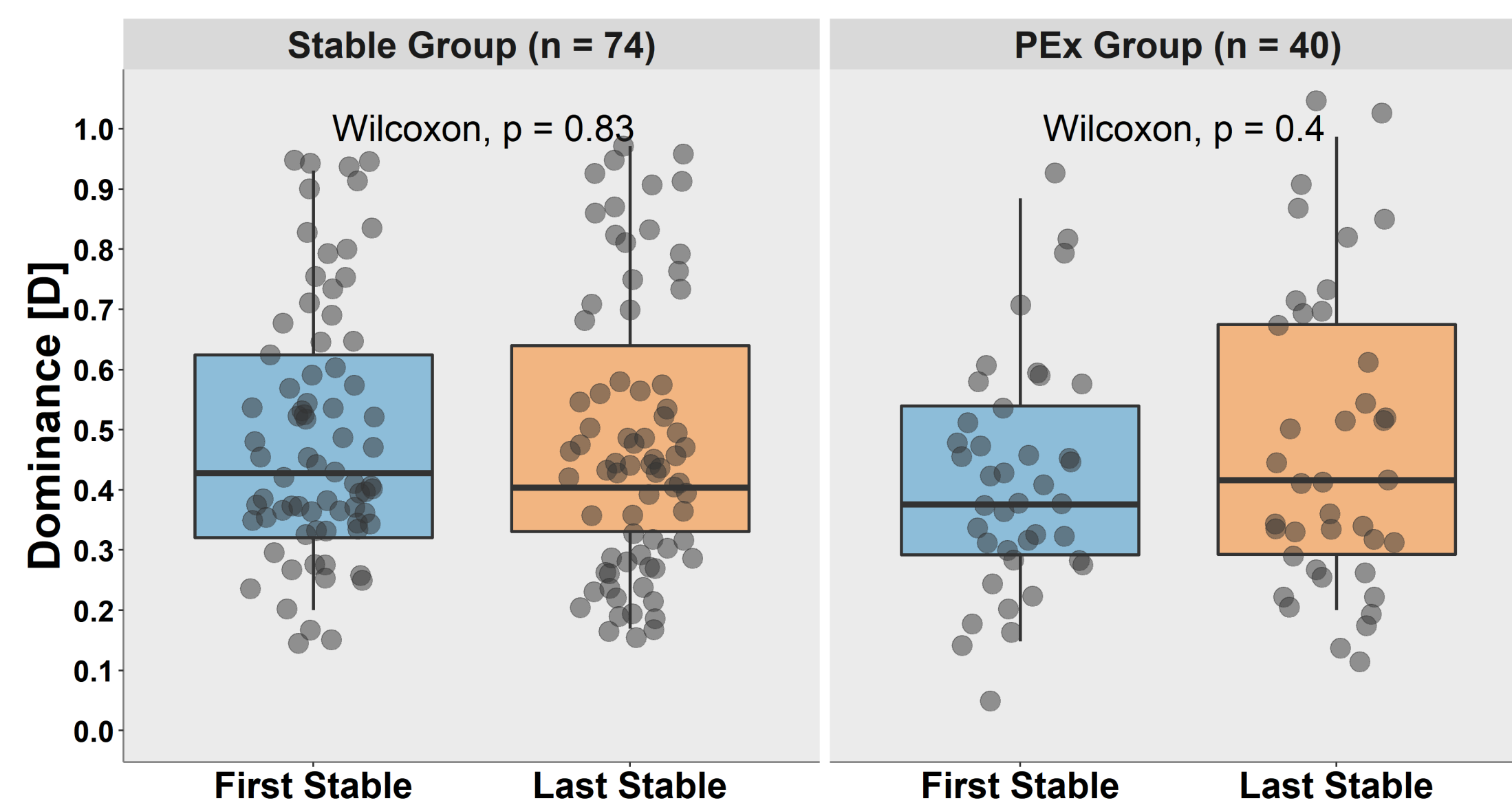**C**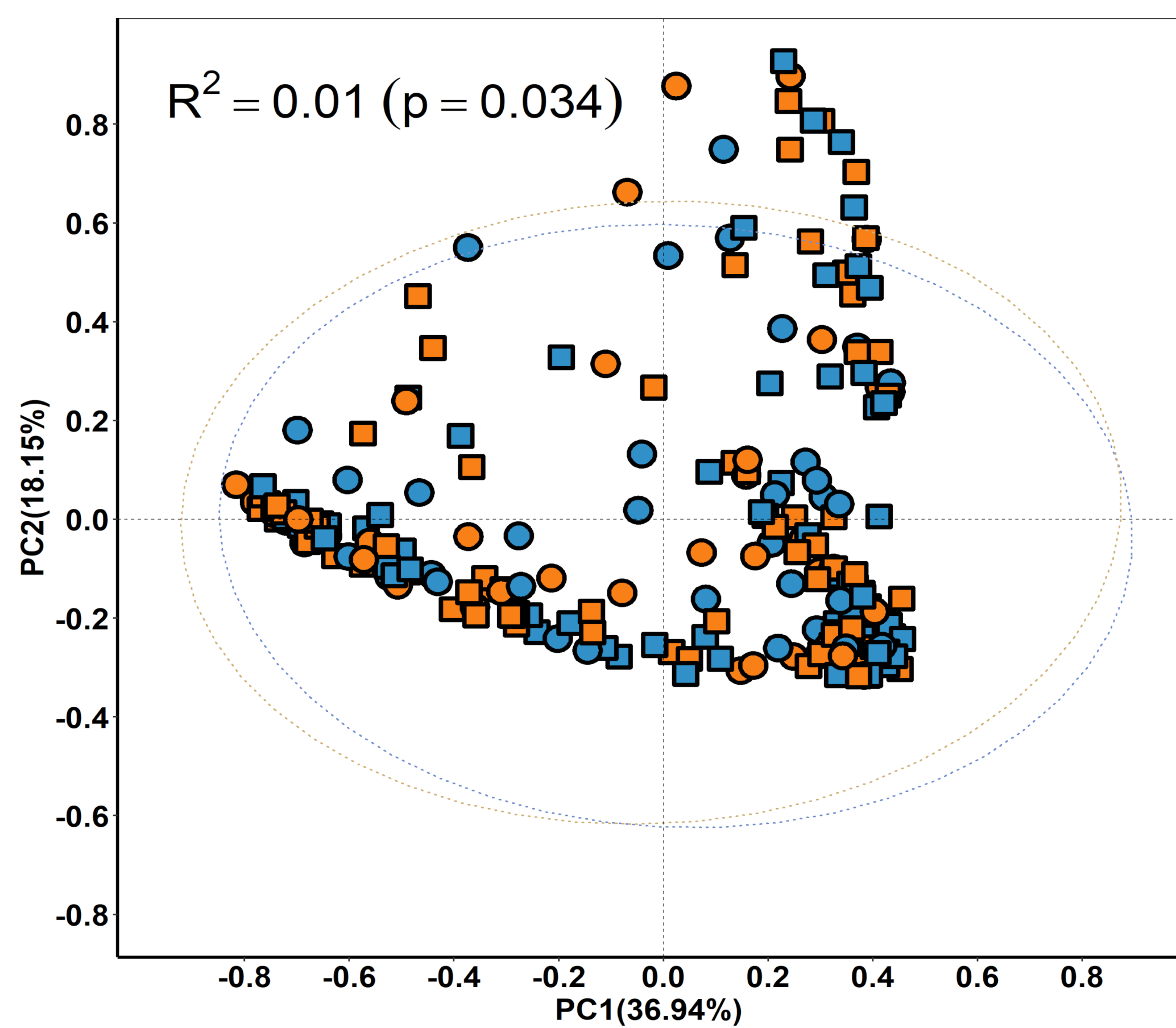

★ First Stable  
 ★ Last Stable  
 □ Stable Group  
 ○ PEx Group

Supplement: Figure S2 [file NIHMS2131163-supplement-Figure_S2.pdf]

Relative Abundance (%)

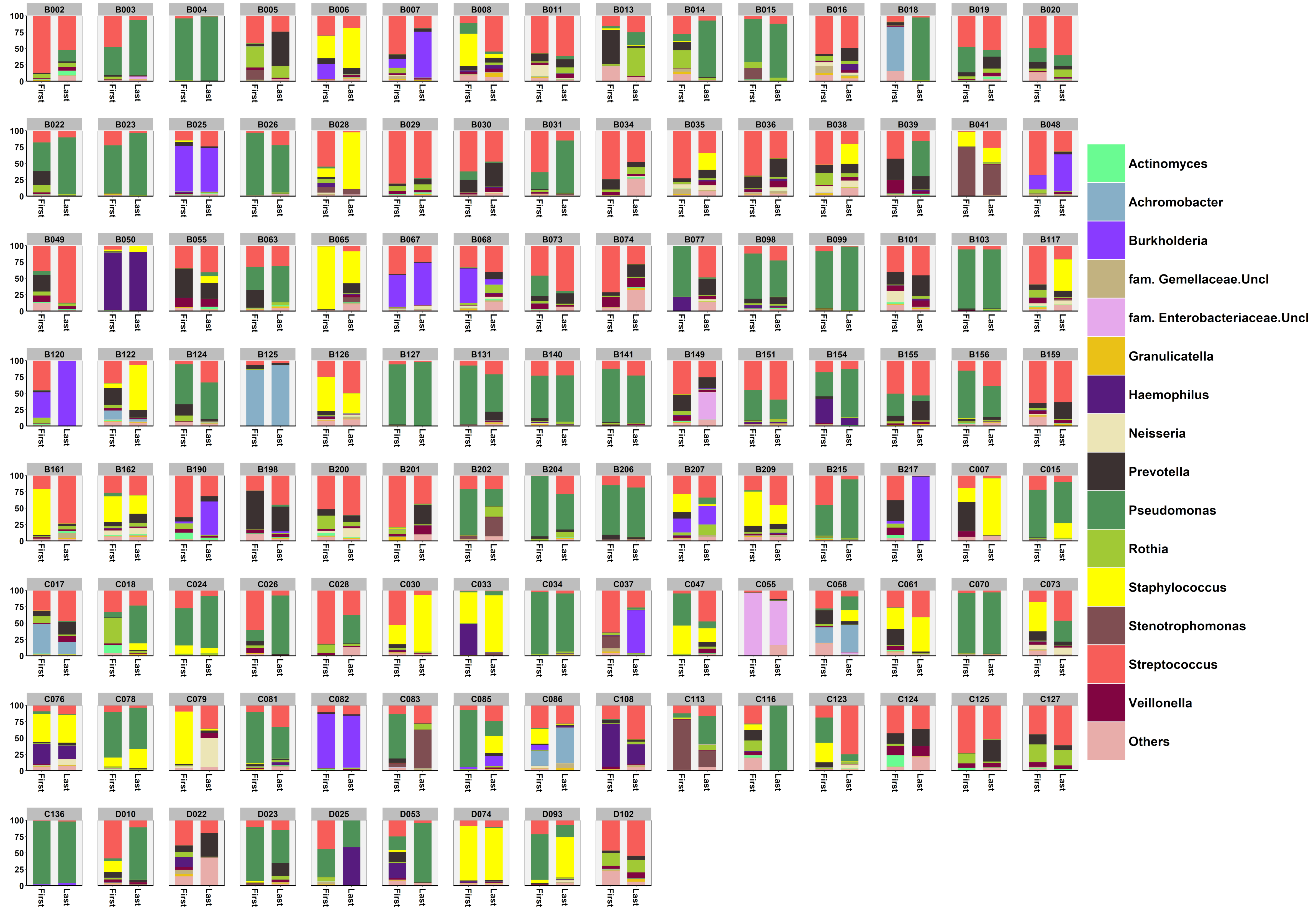

Supplement: Figure S1 [file NIHMS2131163-supplement-Figure_S1.pdf]

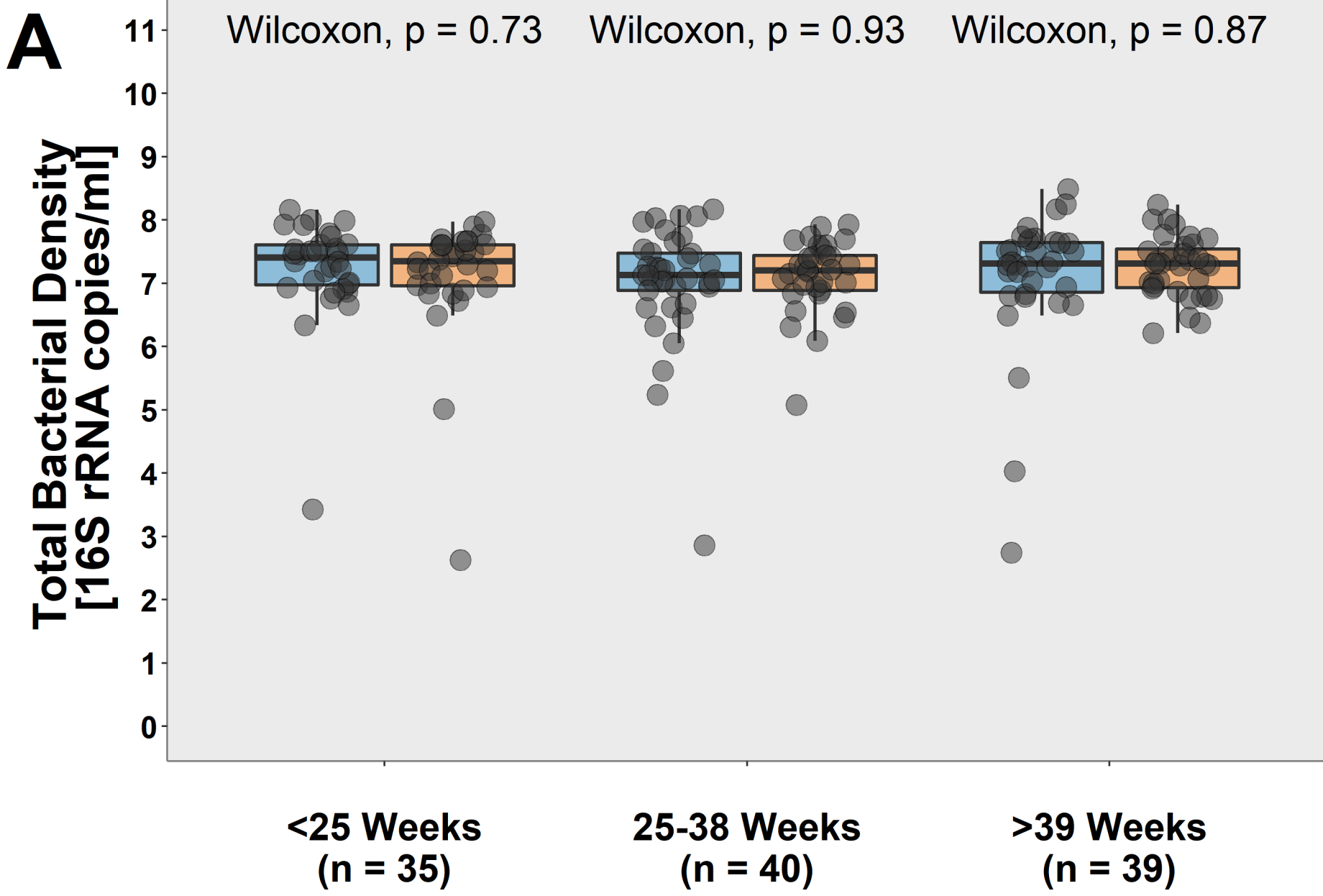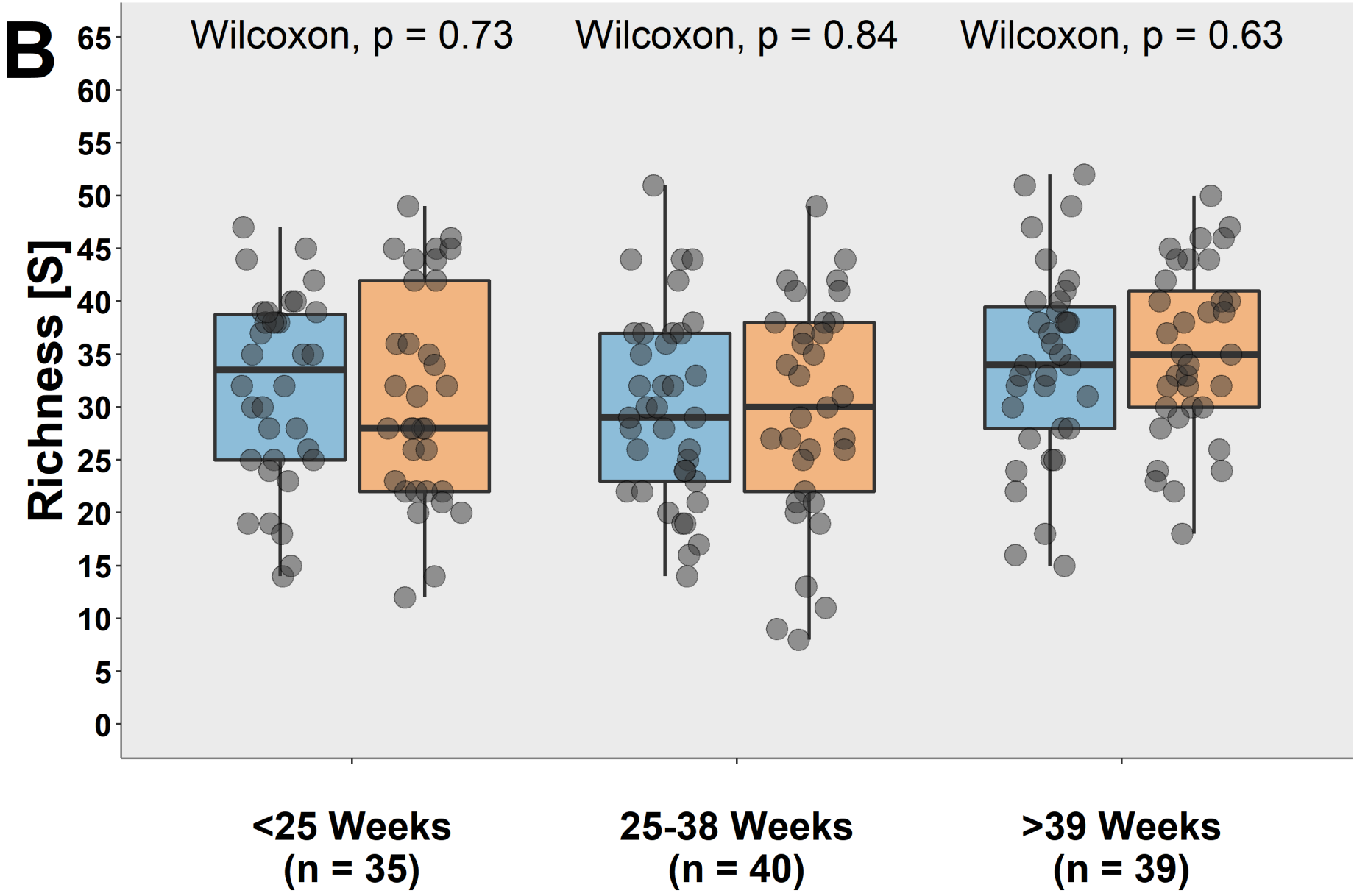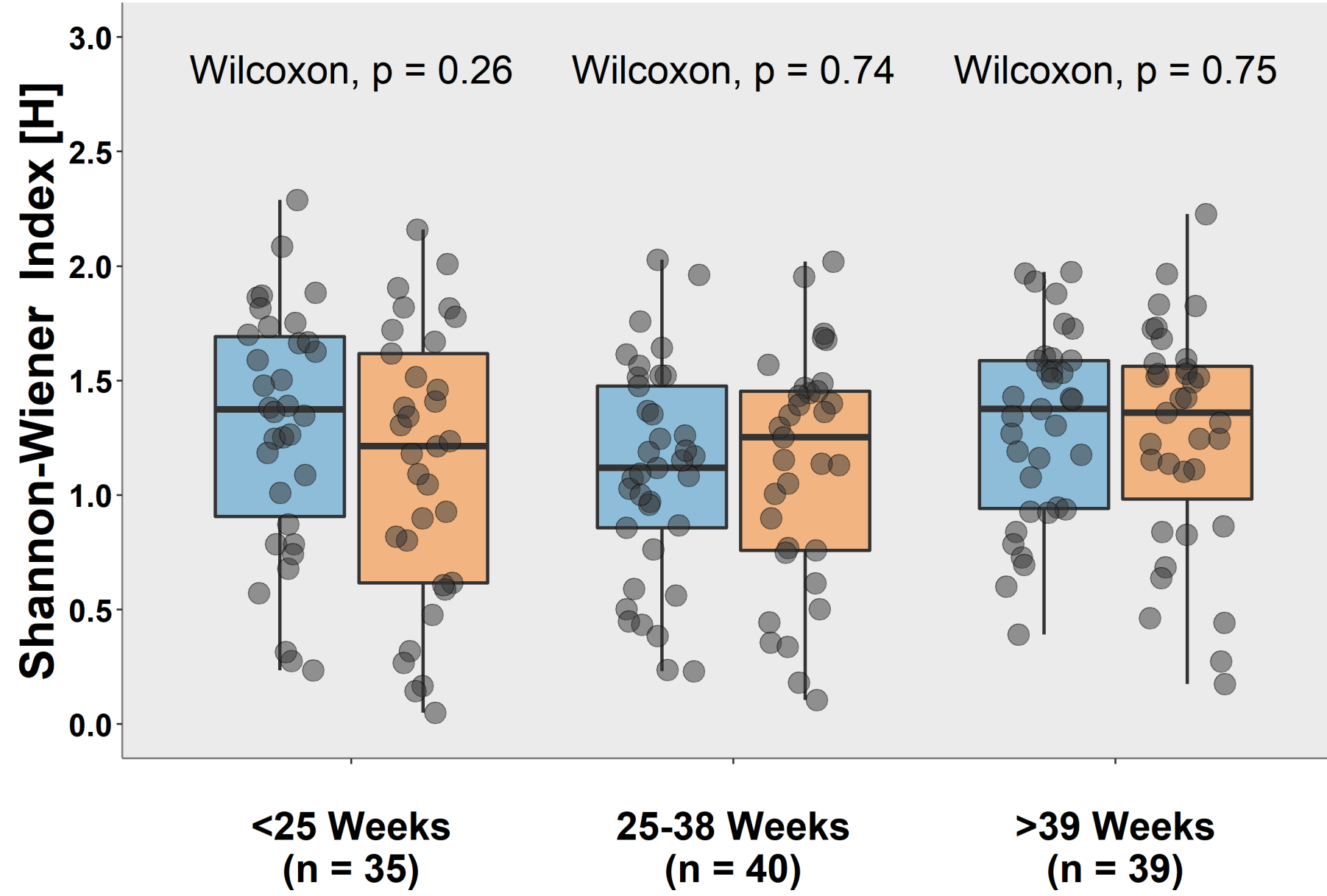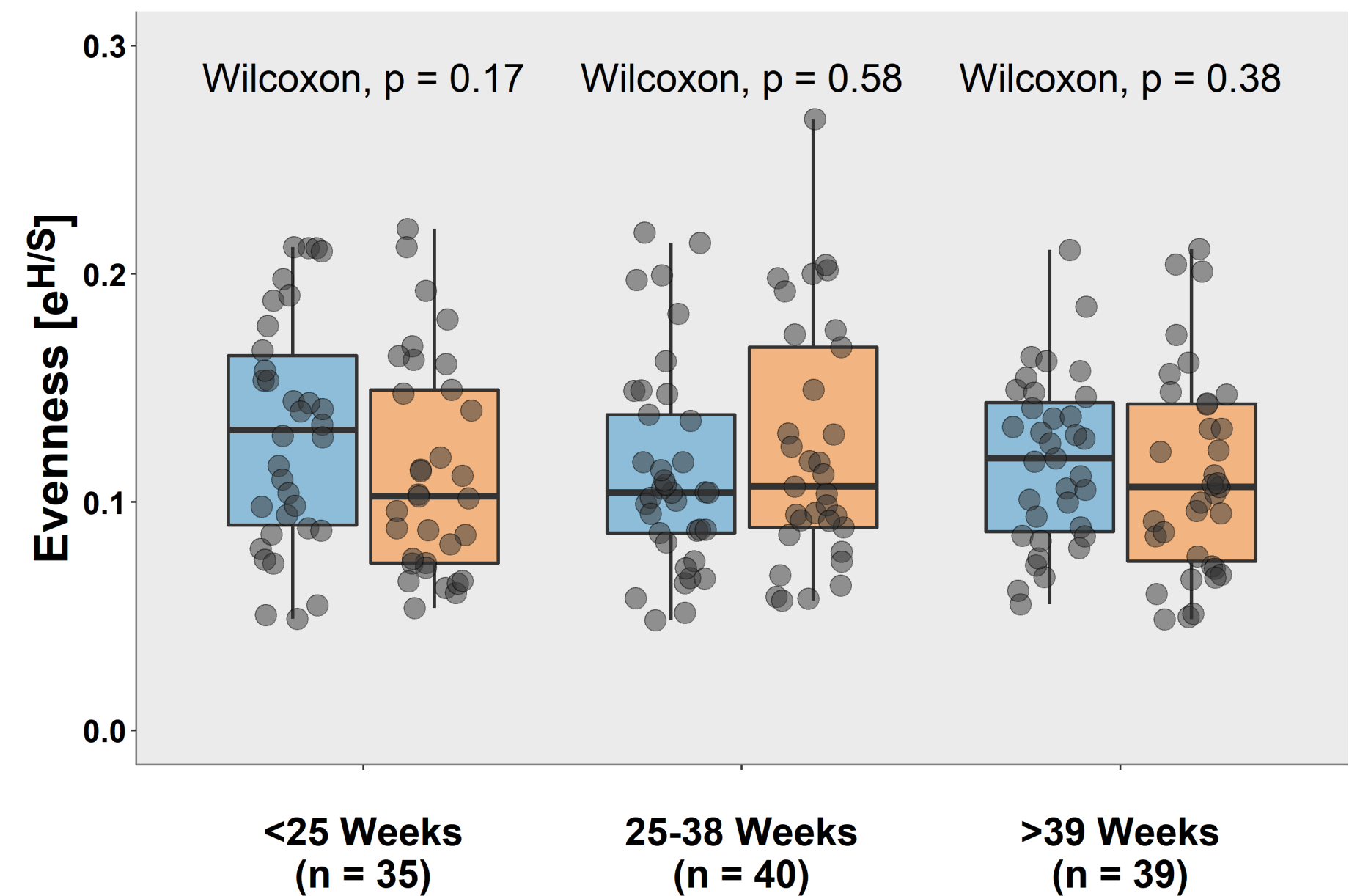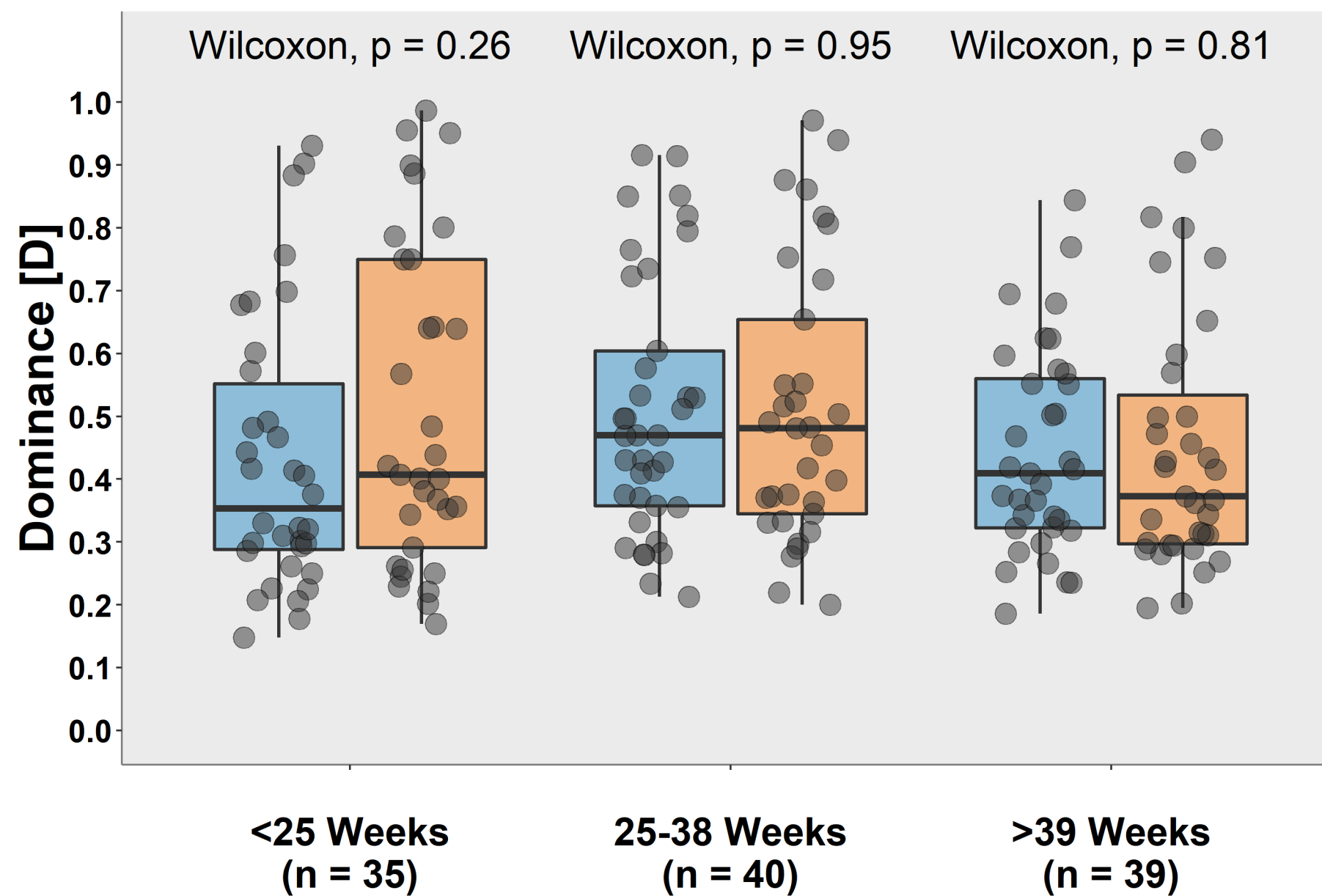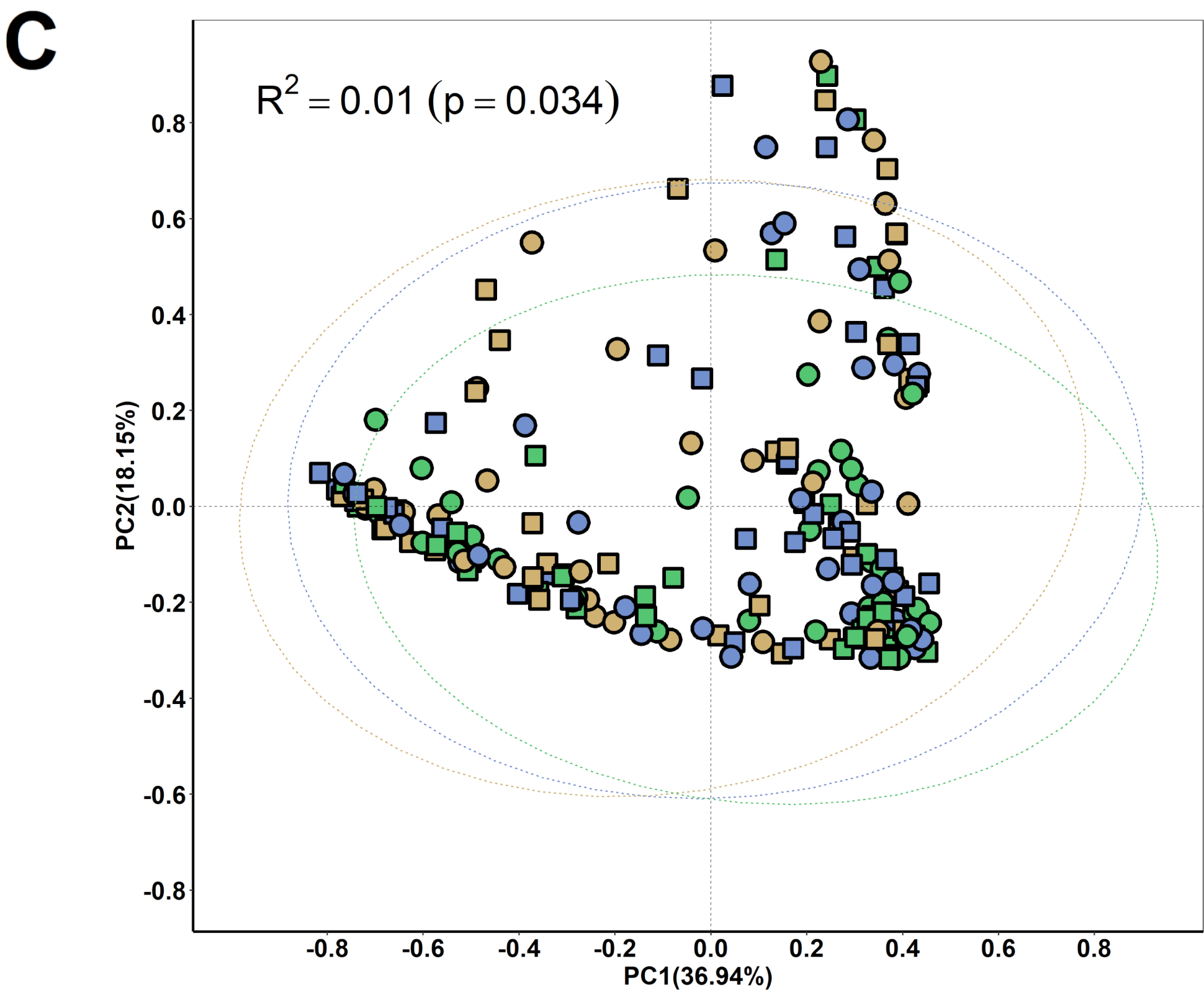

○ First Stable  
□ Last Stable

★ <25 Weeks (n = 35)  
★ 25-38 Weeks (n = 40)  
★ >39 Weeks (n = 39)

Supplement: Figure S3 [file NIHMS2131163-supplement-Figure_S3.pdf]

$R^2 = 0.027$ ;  $p = 0.002$

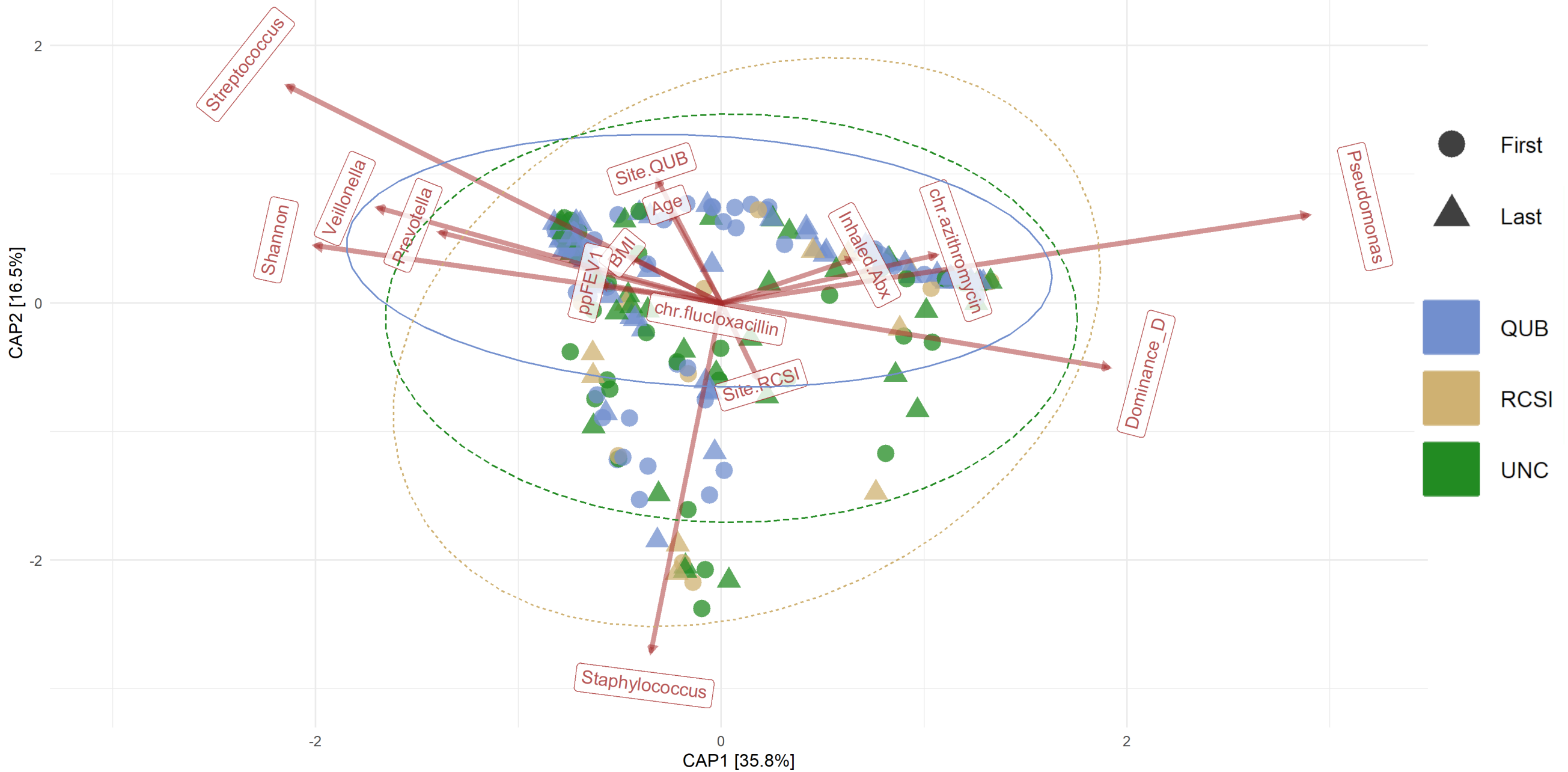

Supplement: Figure S4 [file NIHMS2131163-supplement-Figure_S4.pdf]

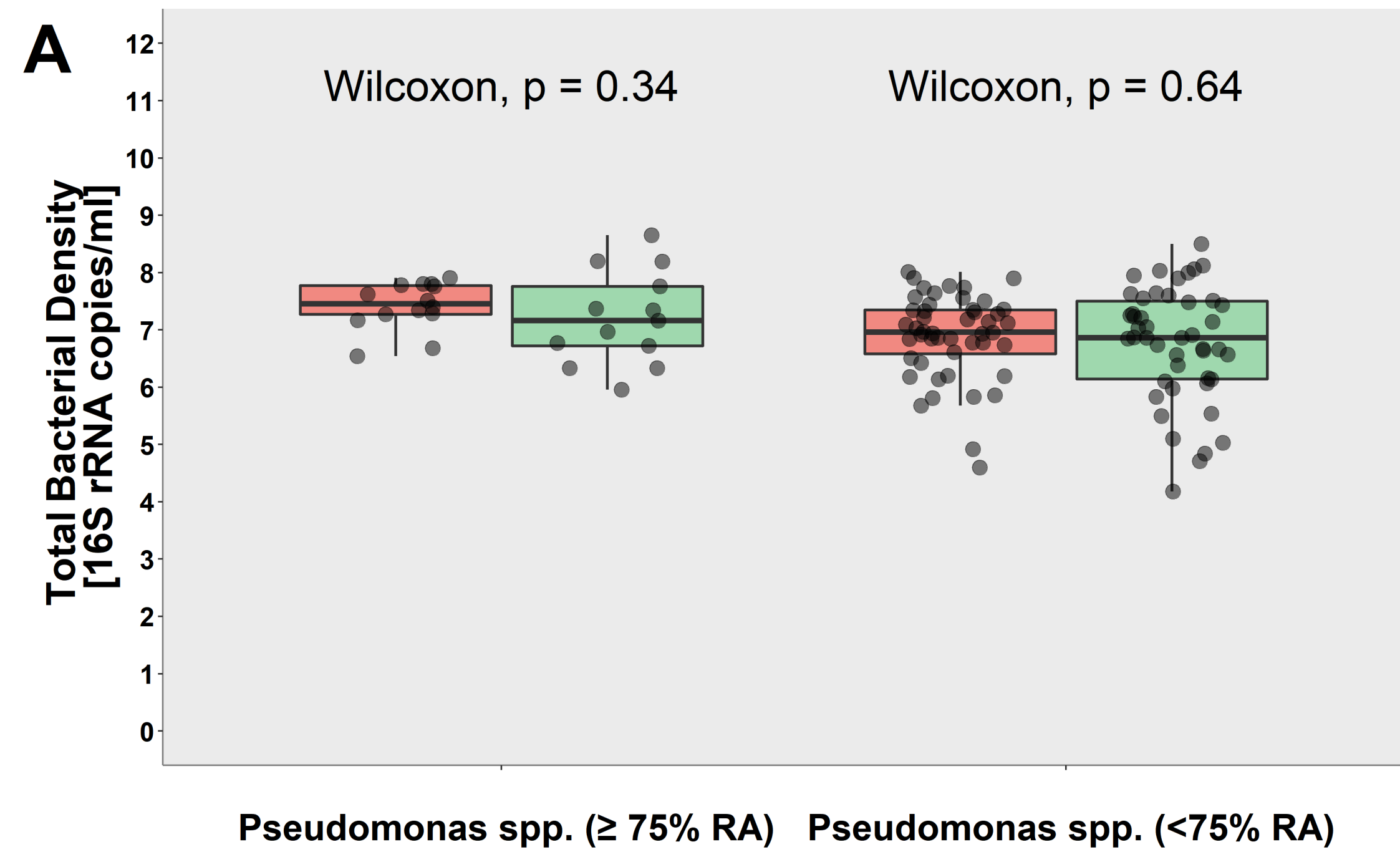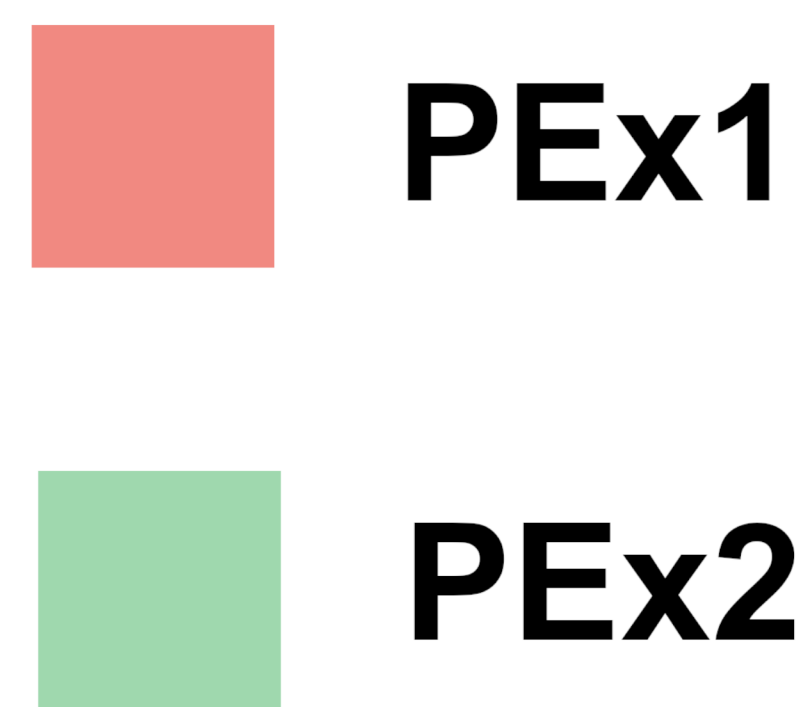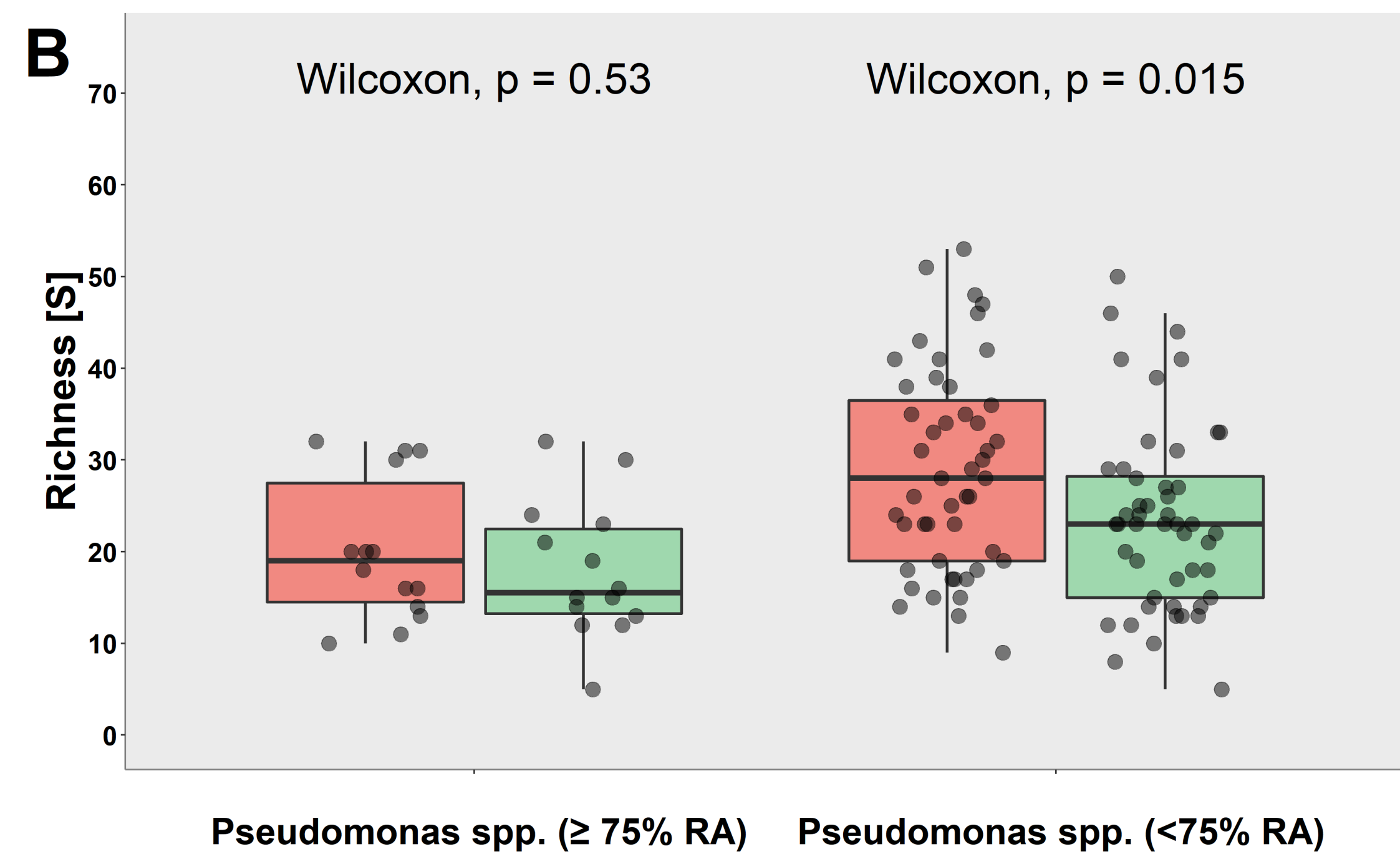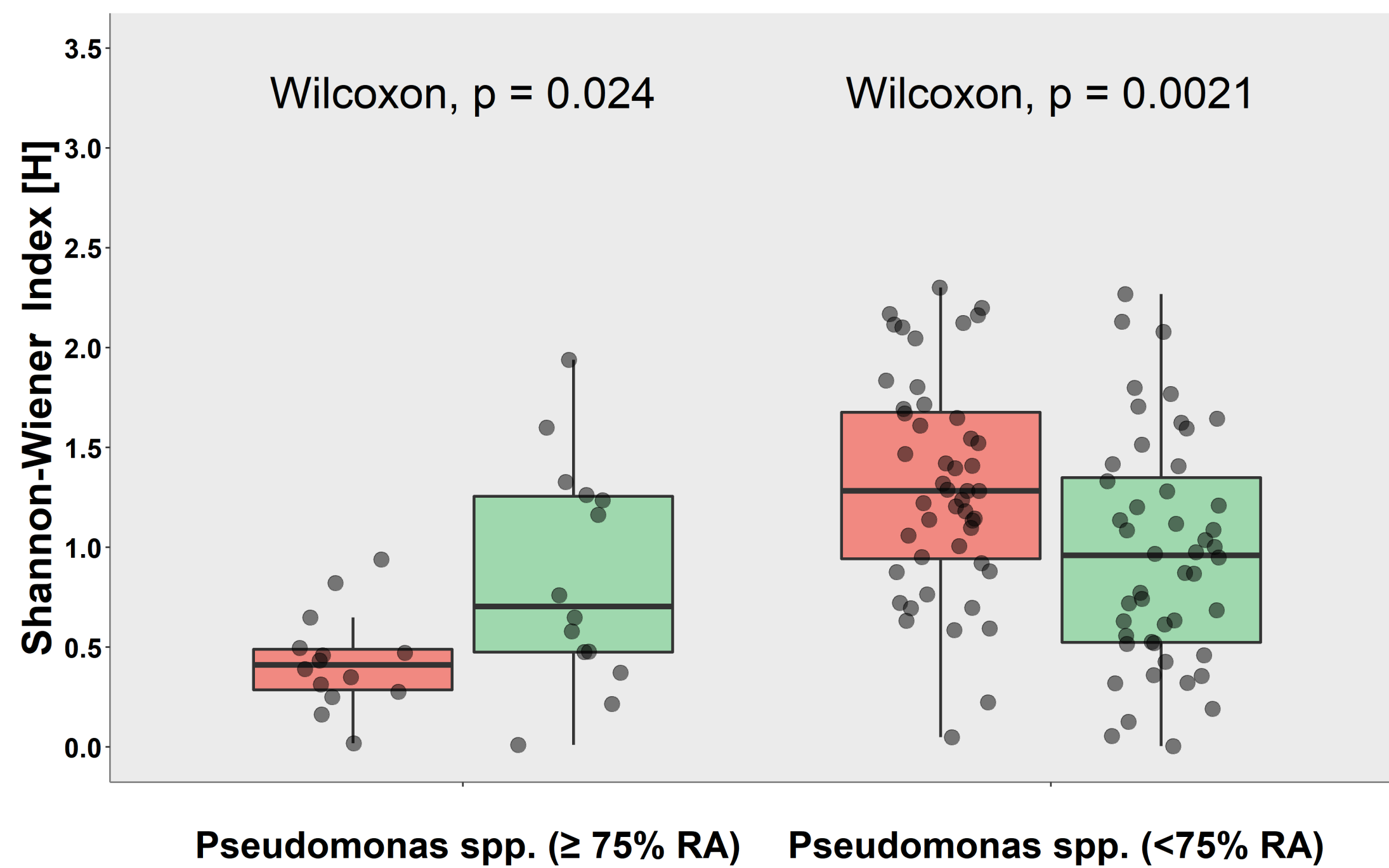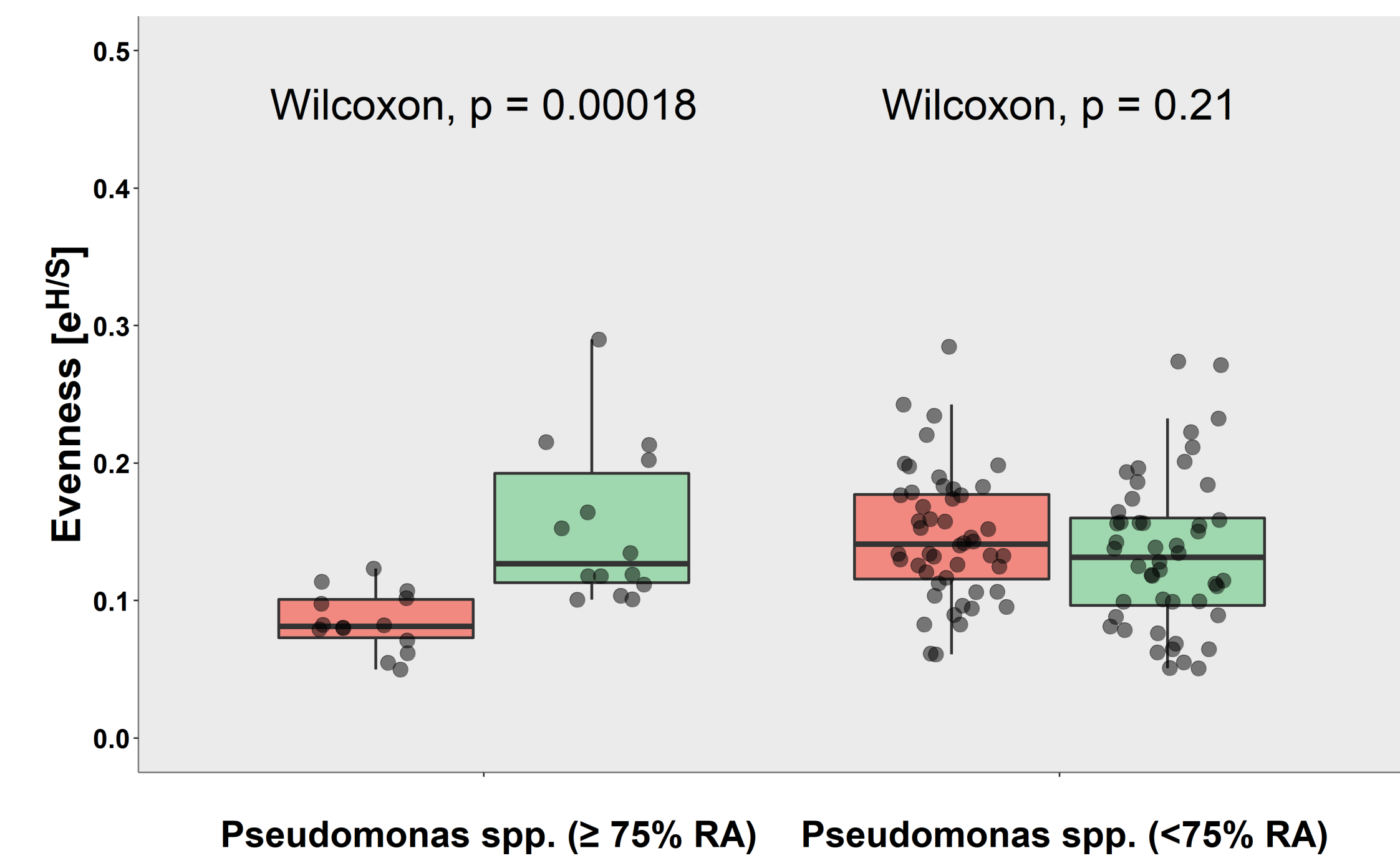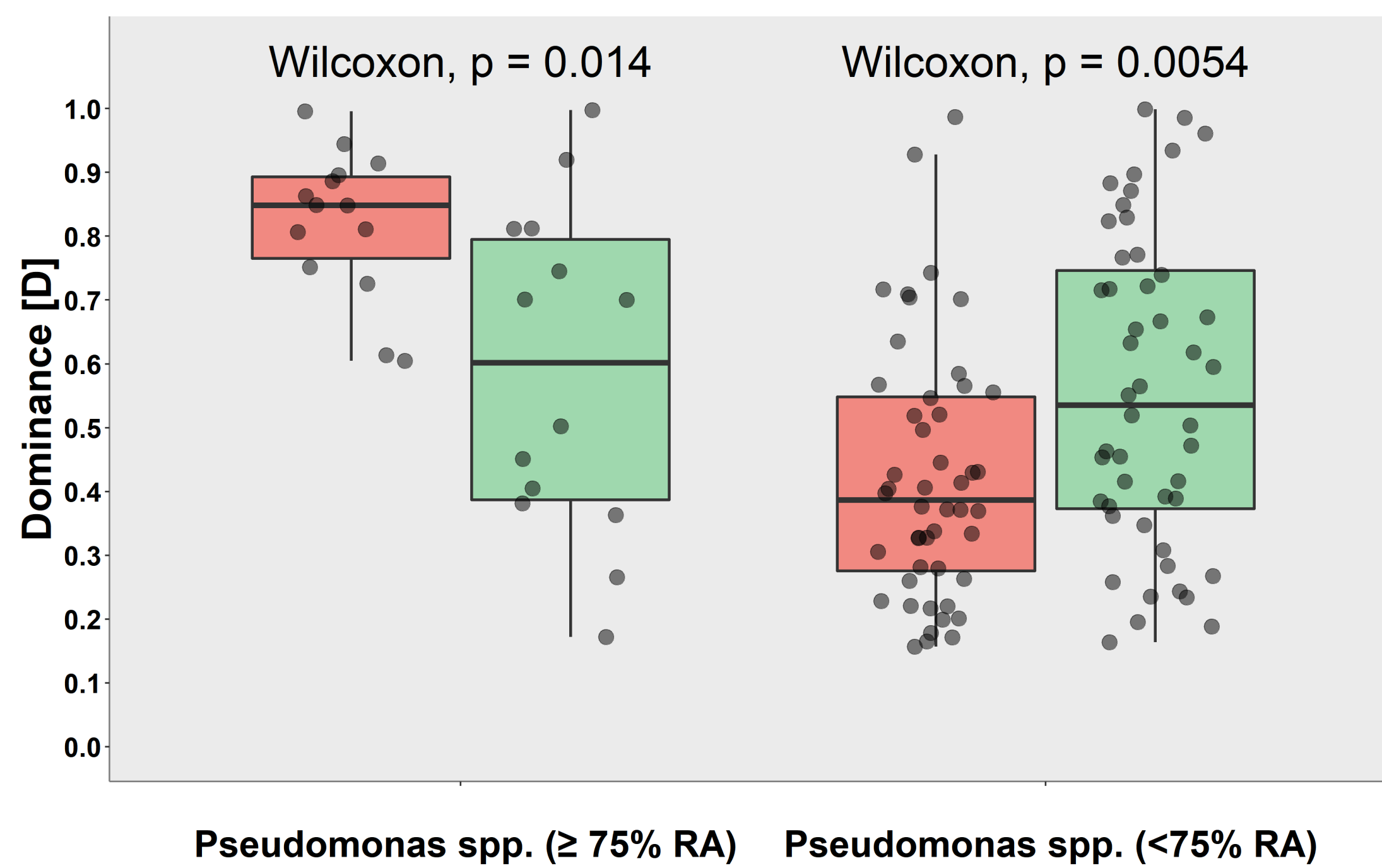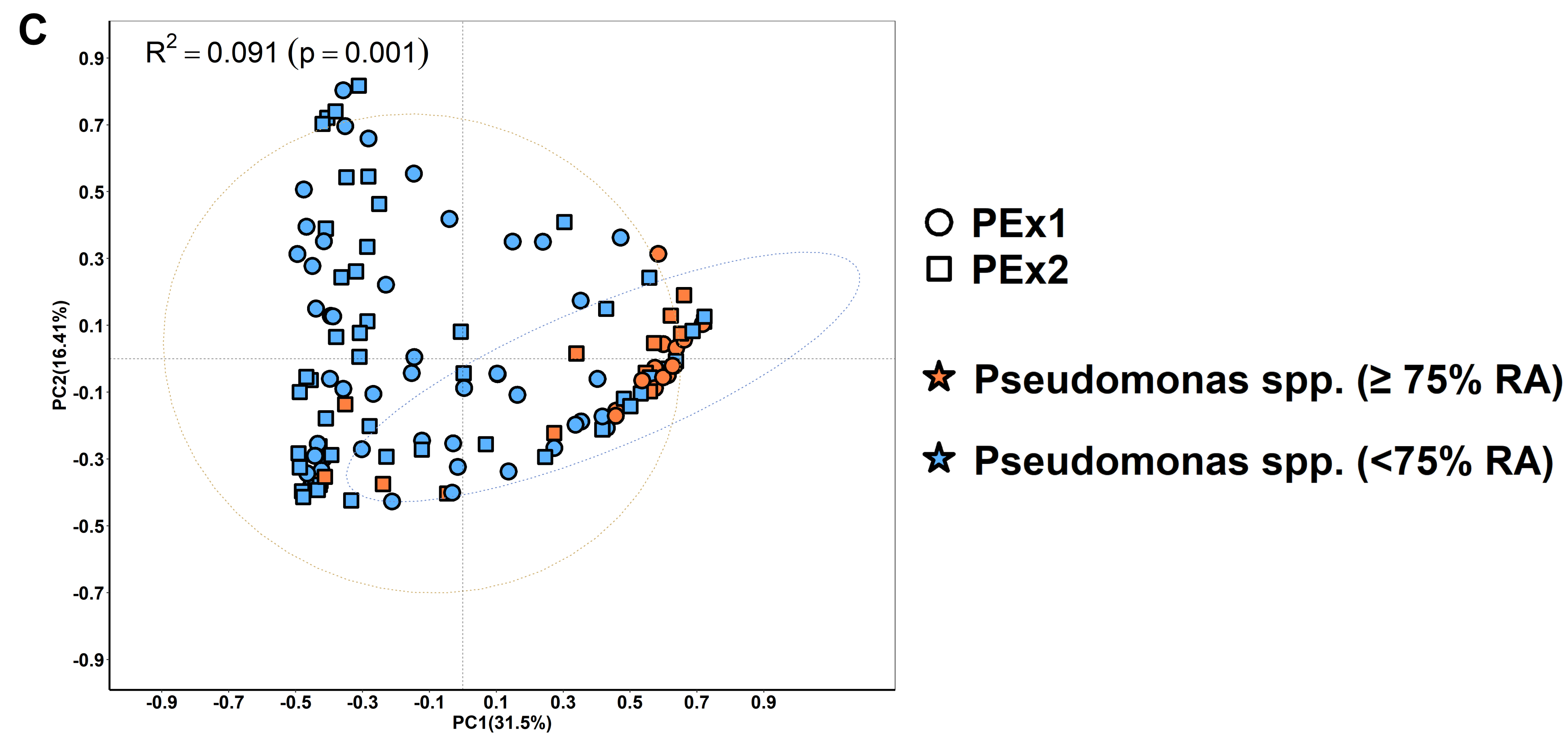

Supplement: Figure S7 [file NIHMS2131163-supplement-Figure_S7.pdf]

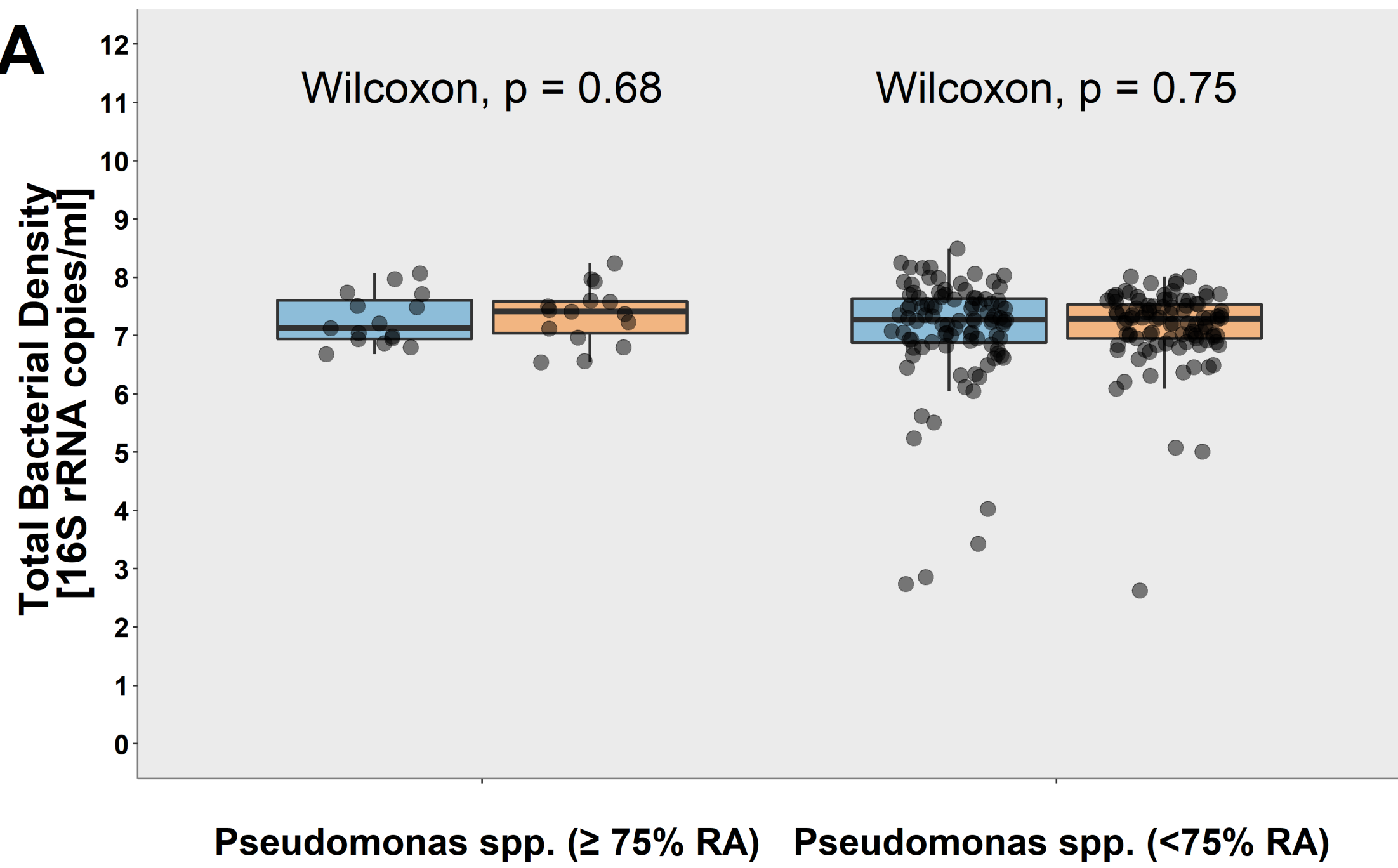

First Stable  
Last Stable

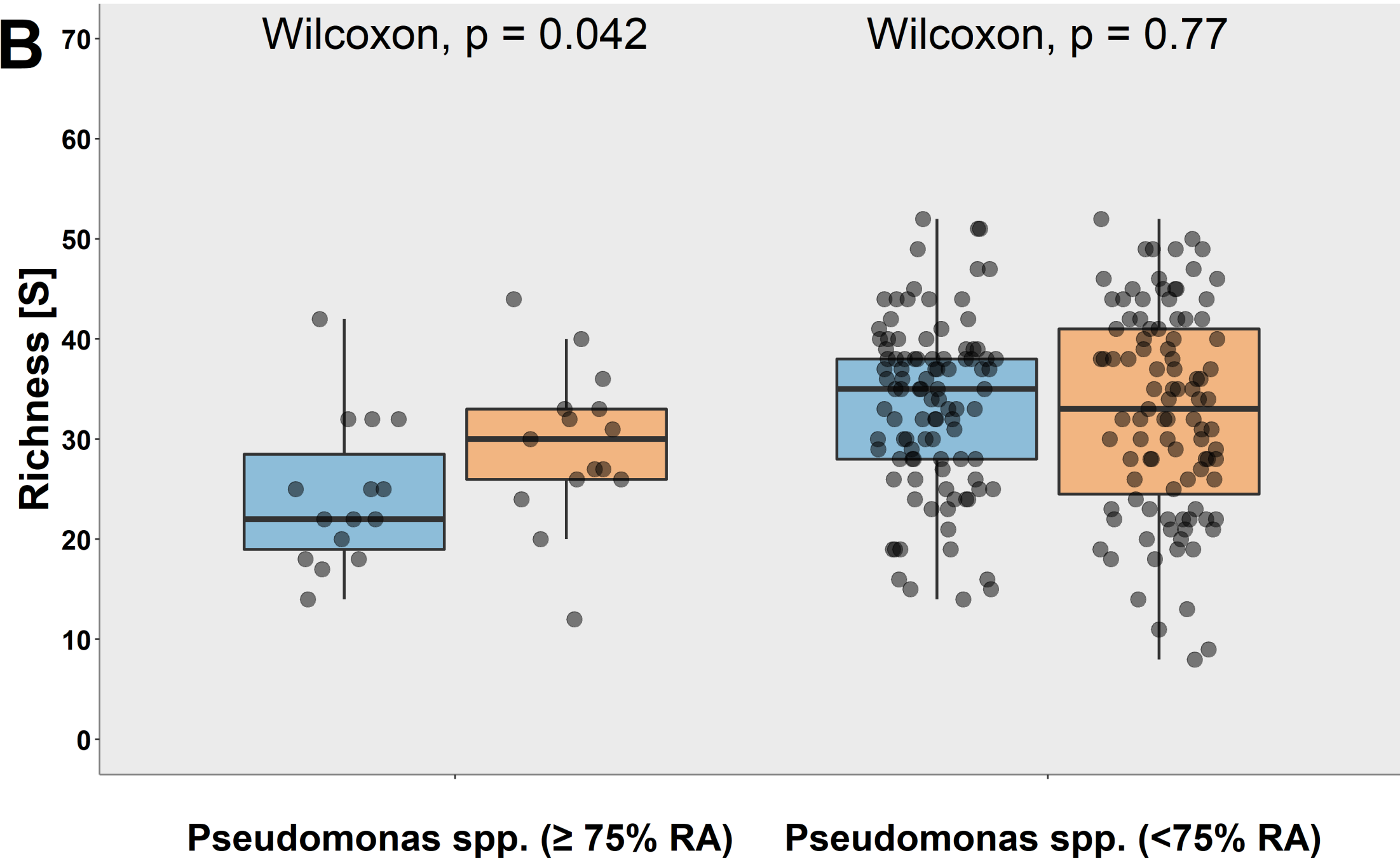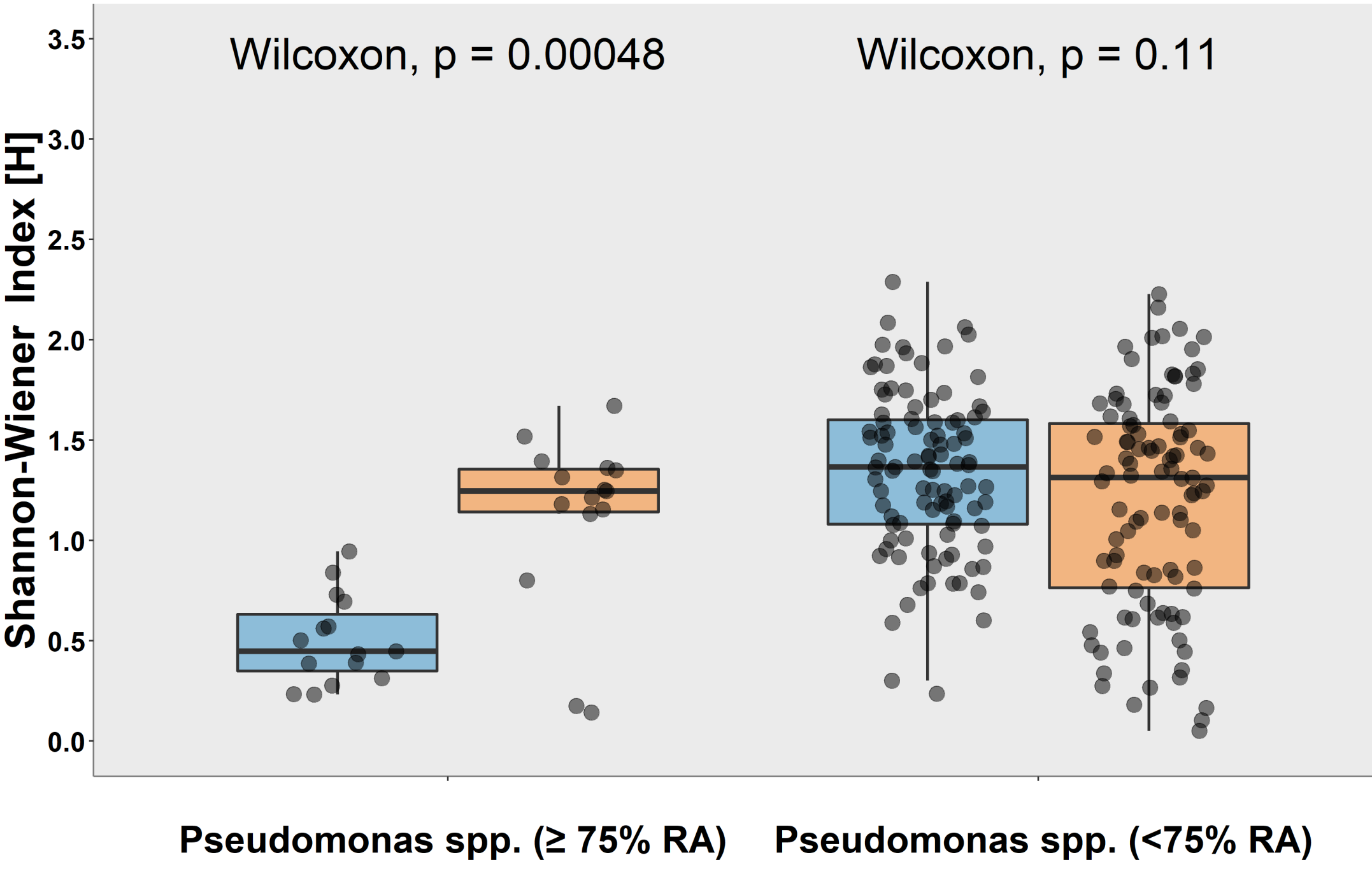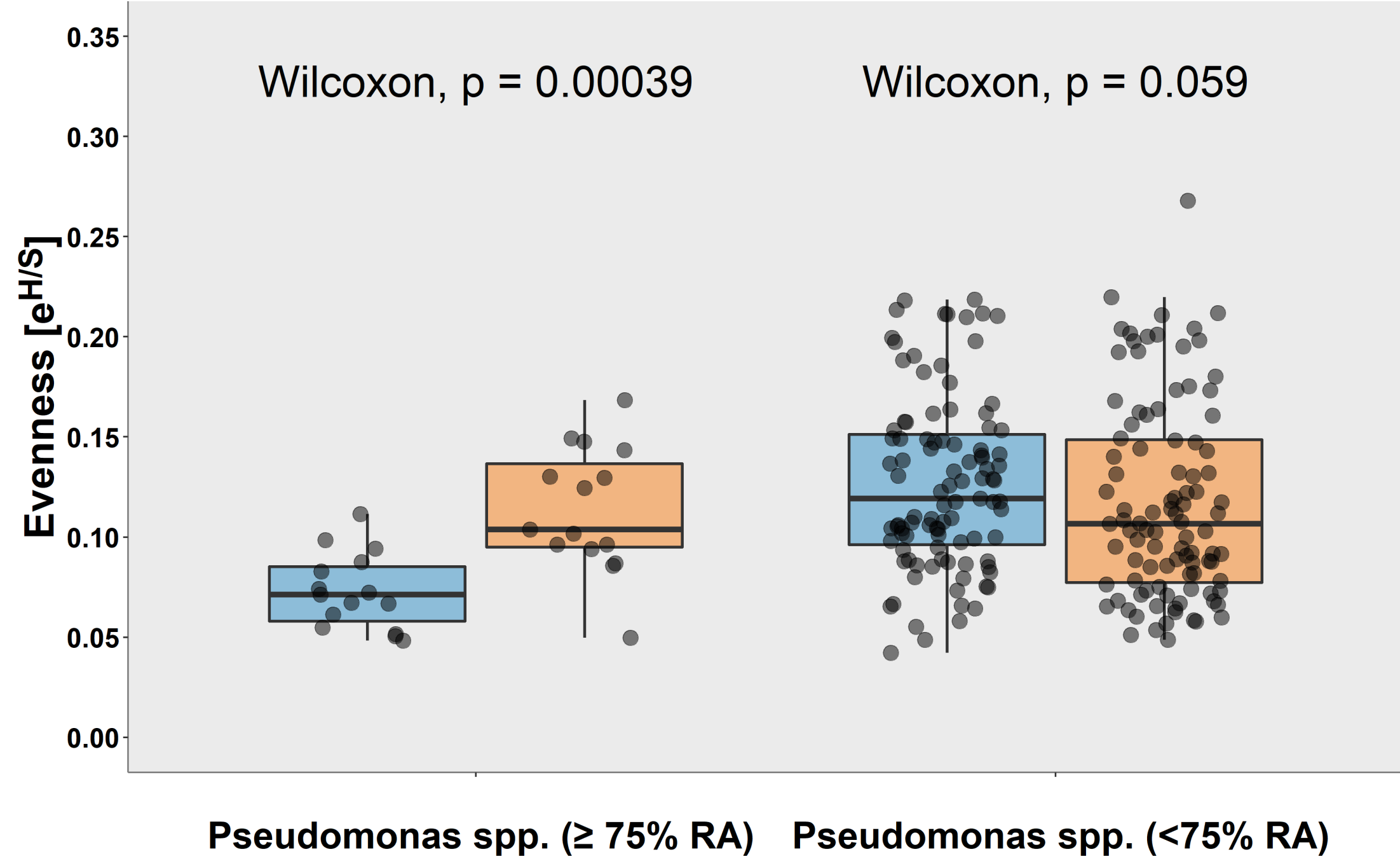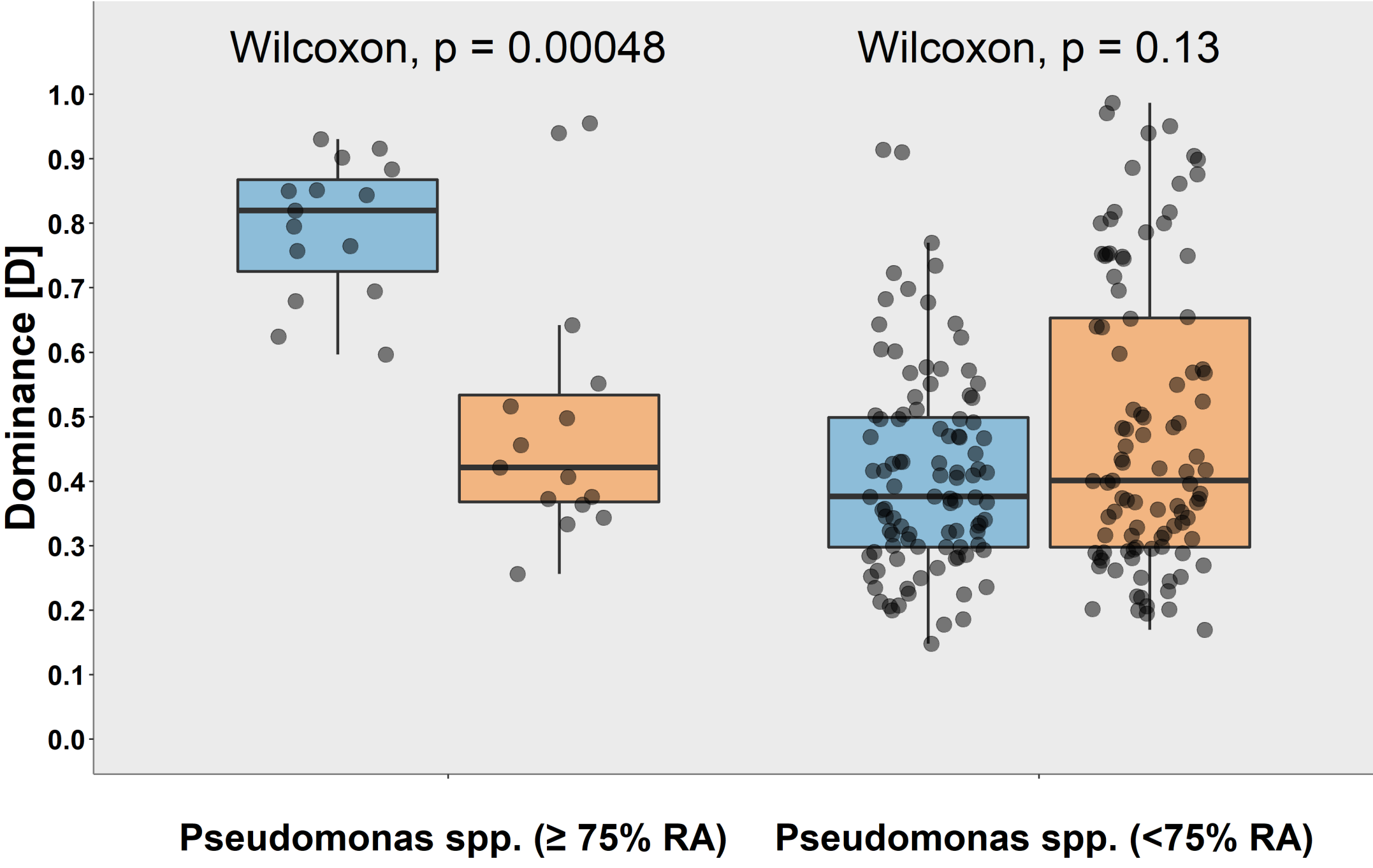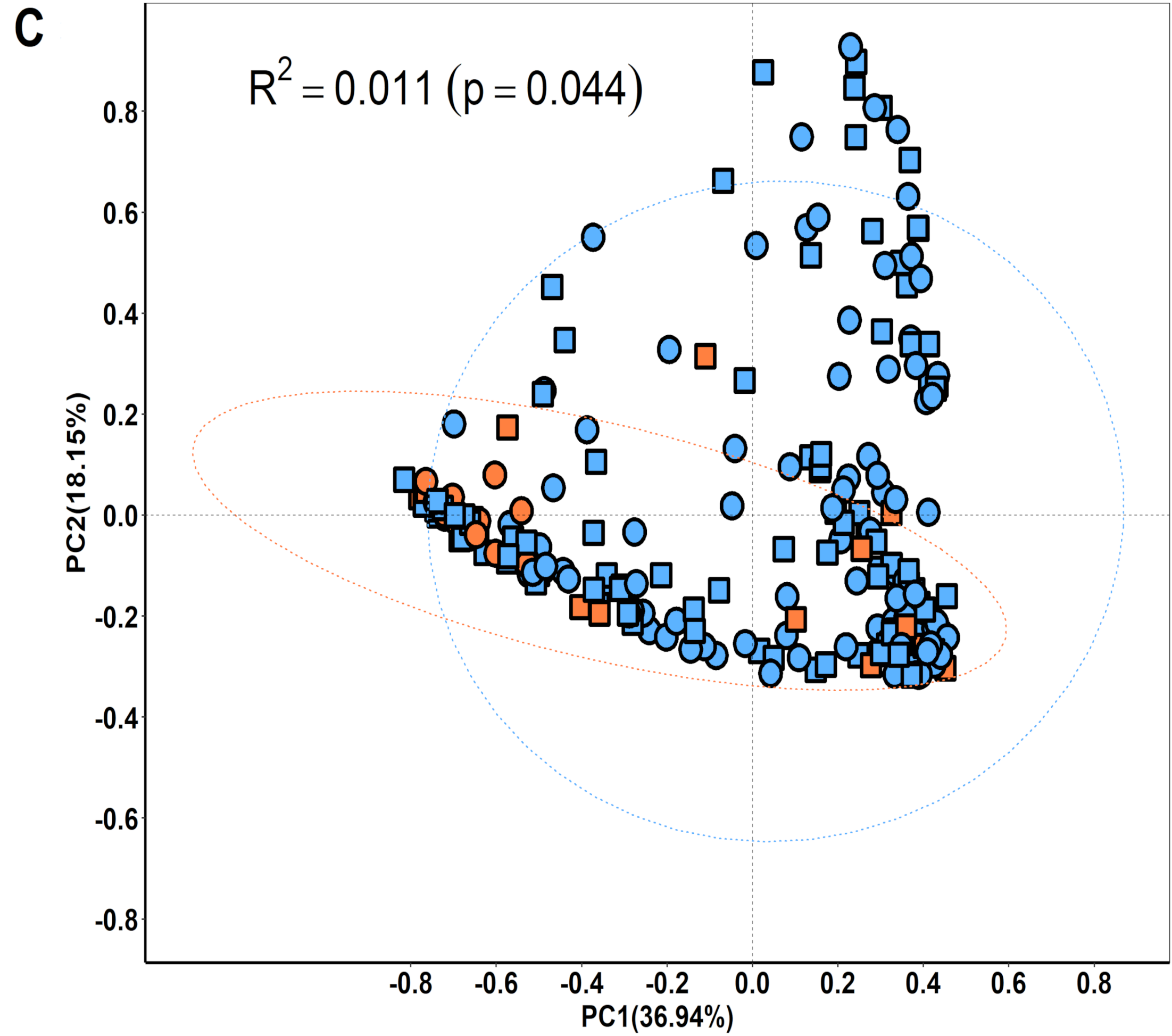

Supplement: Figure S5 [file NIHMS2131163-supplement-Figure_S5.pdf]

**A**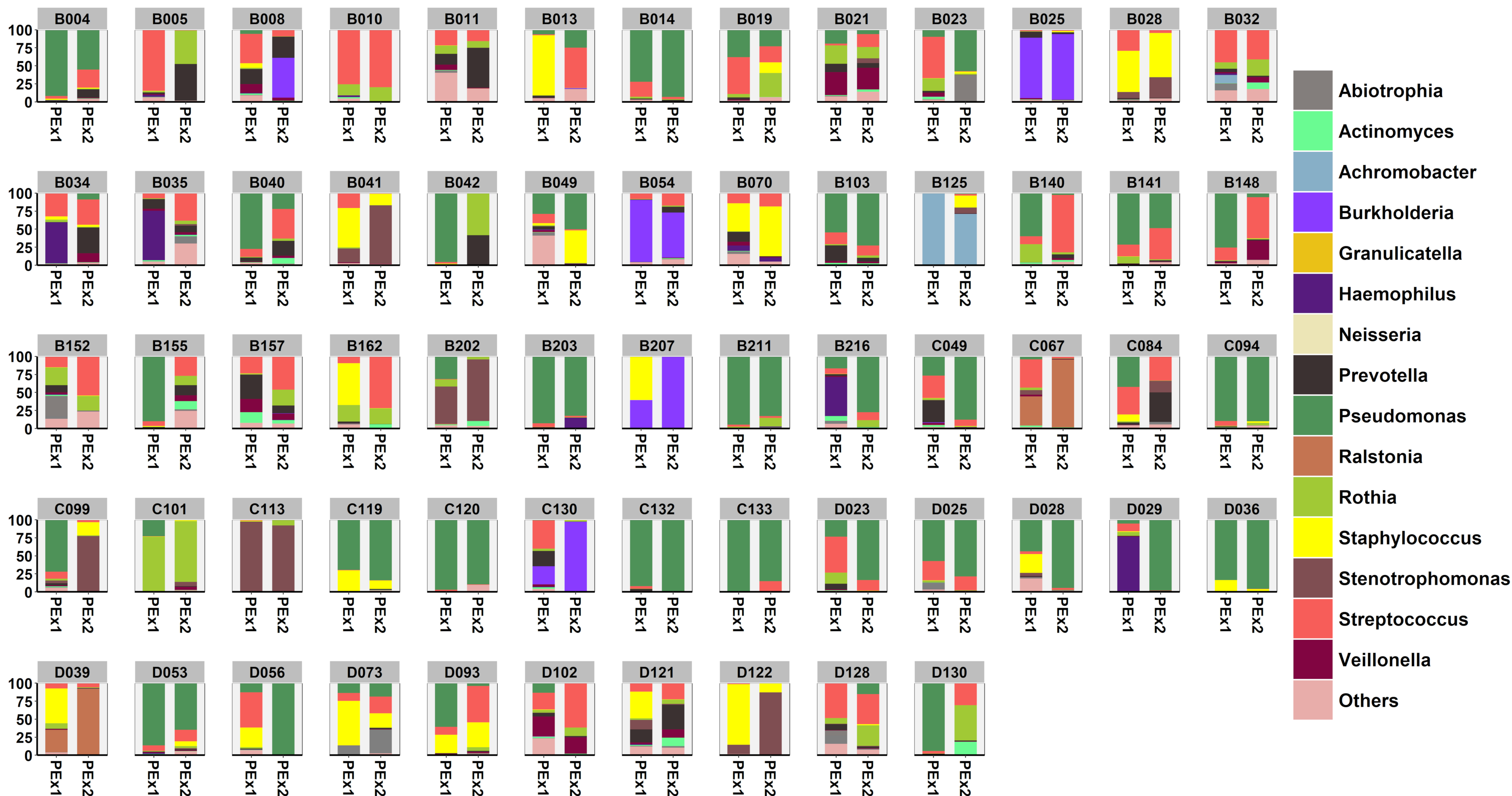**B**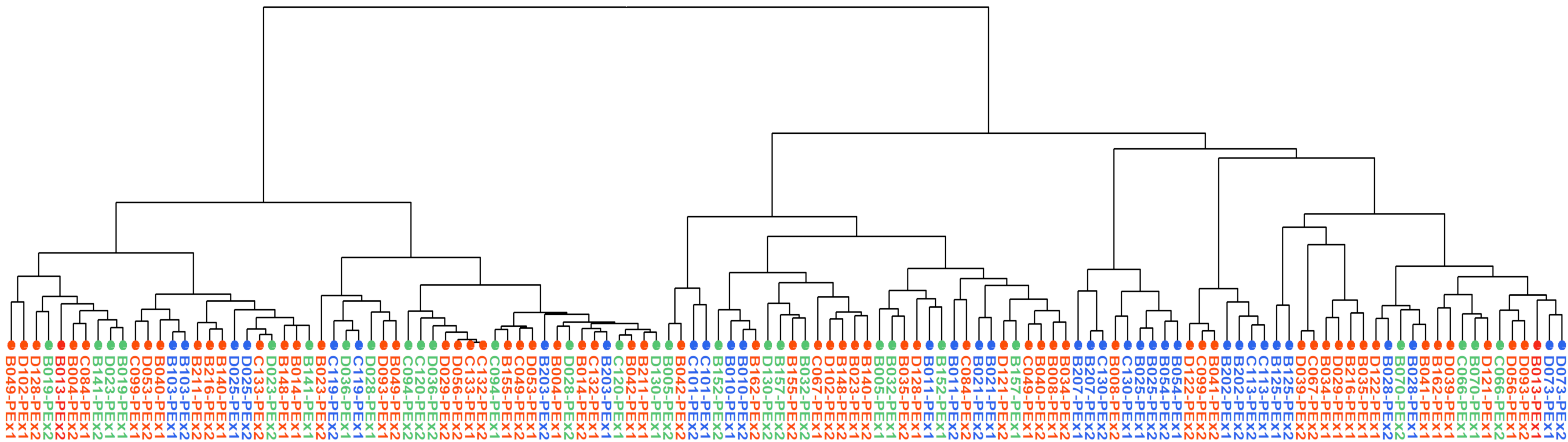

Supplement: Figure S6 [file NIHMS2131163-supplement-Figure_S6.pdf]
